# Plant Extracts and Phytochemicals from the Asteraceae Family with Antiviral Properties

**DOI:** 10.3390/molecules29040814

**Published:** 2024-02-09

**Authors:** Jimena Borgo, Mariel S. Wagner, Laura C. Laurella, Orlando G. Elso, Mariana G. Selener, María Clavin, Hernán Bach, César A. N. Catalán, Augusto E. Bivona, Claudia S. Sepúlveda, Valeria P. Sülsen

**Affiliations:** 1 Instituto de Química y Metabolismo del Fármaco (IQUIMEFA), CONICET-Universidad de Buenos Aires, Junín 956, Piso 2, Buenos Aires C1113AAD, Argentina; jborgo@docente.ffyb.uba.ar (J.B.); c.laurella@docente.ffyb.uba.ar (L.C.L.); 2 Facultad de Farmacia y Bioquímica, Universidad de Buenos Aires, Junín 956, Piso 2, Buenos Aires C1113AAD, Argentina; orlando.elso@gmail.com (O.G.E.); mgsmarian@gmail.com (M.G.S.); mclavin@ffyb.uba.ar (M.C.); 3 Laboratorio de Estrategias Antivirales, Departamento de Química Biológica, Facultad de Ciencias Exactas y Naturales, Universidad de Buenos Aires, Int. Güiraldes 2160, Piso 4, Buenos Aires C1428EGA, Argentina; mariel.wagner22@gmail.com; 4 Unidad de Microanálisis y Métodos Físicos Aplicados a Química Orgánica (UMYMFOR), Facultad de Ciencias Exactas y Naturales, CONICET-Universidad de Buenos Aires, Ciudad Universitaria, Pabellón 2, Piso 3, Buenos Aires C1428EGA, Argentina; 5 Instituto Nacional de Tecnología Agropecuaria (INTA) Gobernador Guillermo Udaondo 1695 Estación Experimental Agropecuaria Área Metropolitana de Buenos Aires, EEA AMBA Udaondo, Villa Udaondo B1713AAW, Buenos Aires Province, Argentina; bach.hernan@inta.gob.ar; 6 Instituto de Química Orgánica, Facultad de Bioquímica, Química y Farmacia, Universidad Nacional de Tucumán, Ayacucho 471, San Miguel de Tucumán T4000INI, Tucumán Province, Argentina; cancatalan@gmail.com; 7 Instituto de Estudios de la Inmunidad Humoral Prof. Ricardo A. Margni (IDEHU), CONICET-Universidad de Buenos Aires, Junín 956, Piso 4, Buenos Aires C1113AAD, Argentina; aebivona@ffyb.uba.ar; 8 Instituto de Investigaciones en Microbiología y Parasitología Médica (IMPaM), CONICET-Universidad de Buenos Aires, Paraguay 2155, Piso 13, Buenos Aires C1121ABG, Argentina; 9 Instituto de Química Biológica de la Facultad de Ciencias Exactas y Naturales (IQUIBICEN), CONICET-Universidad de Buenos Aires, Int. Güiraldes 2160, Piso 4, Buenos Aires C1428EGA, Argentina

**Keywords:** Asteraceae, dengue, plant extract, terpenoids, phenolic compounds, sesquiterpene lactones, flavonoids, antiviral

## Abstract

Asteraceae (Compositae), commonly known as the sunflower family, is one of the largest plant families in the world and includes several species with pharmacological properties. In the search for new antiviral candidates, an in vitro screening against dengue virus (DENV) was performed on a series of dichloromethane and methanolic extracts prepared from six Asteraceae species, including *Acmella bellidioides*, *Campuloclinium macrocephalum*, *Grindelia pulchella*, *Grindelia chiloensis*, *Helenium radiatum*, and *Viguiera tuberosa*, along with pure phytochemicals isolated from Asteraceae: mikanolide (**1**), eupatoriopicrin (**2**), eupahakonenin B (**3**), minimolide (**4**), estafietin (**5**), 2-oxo-8-deoxyligustrin (**6**), santhemoidin C (**7**), euparin (**8**), jaceidin (**9**), nepetin (**10**), jaceosidin (**11**), eryodictiol (**12**), eupatorin (**13**), and 5-demethylsinensetin (**14**). Results showed that the dichloromethane extracts of *C. macrocephalum* and *H. radiatum* and the methanolic extracts prepared from *C. macrocephalum* and *G. pulchella* were highly active and selective against DENV-2, affording EC_50_ values of 0.11, 0.15, 1.80, and 3.85 µg/mL, respectively, and SIs of 171.0, 18.8, >17.36, and 64.9, respectively. From the pool of phytochemicals tested, compounds **6**, **7**, and **8** stand out as the most active (EC_50_ = 3.7, 3.1, and 6.8 µM, respectively; SI = 5.9, 6.7, and >73.4, respectively). These results demonstrate that Asteraceae species and their chemical constituents represent valuable sources of new antiviral molecules.

## 1. Introduction

Asteraceae (Compositae) represents one of the largest plant families, encompassing over 600 genera and 25,000 species worldwide. Several well-known species, such as sunflower, dahlias, daisy, lettuce, chicory, and coreopsis, belong to this family, popularly known as the sunflower family. The Asteraceae family is distributed worldwide and can be found in a wide range of natural habitats, although most of the species are frequently found in tropical and subtropical areas [1]. Plants from this family have been cultivated for their nutritional value, medicinal uses, and ornamental purposes. Members of this family have exhibited numerous pharmacological activities, including anti-viral, anti-diabetic, anti-tumor, anti-nociceptive, anti-inflammatory, hepatoprotective, anti-parasitic, anti-malarial, and wound-healing properties, among others [2]. These medicinal properties are attributed to the presence of bioactive compounds from which polyphenols such as flavonoids and terpenoids, including sesquiterpene lactones, stand out [3].

Dengue fever is a disease caused by infection with dengue virus (DENV), which has four serotypes, and it is transmitted by infected female mosquitoes of the *Aedes* genus. The World Health Organization considers it one of the twenty neglected tropical diseases since it is prevalent in tropical and subtropical climates throughout the world, especially in urban and semi-urban areas. Currently, half of the world’s population is at risk of contracting this virus, and every year, between 100 and 400 million infections occur [4]. Dengue cases in the Americas have increased in the last four decades, going from 1.5 million cumulative cases in the 1980s to 16.2 million in the 2010–2019 decade [5]. According to the epidemiological bulletin from the National Ministry of Health in Argentina, from the epidemiological week (EW) 31/2023 to EW 1/2024, 17,540 cases of dengue fever were recorded, mostly of autochthonous origin, with an incidence of 38 cases/100,000 inhabitants. Specifically in the northeast region of Argentina (NEA region), the incidence amounts to 399 cases/100,000 inhabitants. Eight provinces so far have five or more cases of dengue without travel history in their territory this season, and twenty-three of the twenty-four jurisdictions have presented imported cases. In this season, three serotypes circulating in the country have so far been identified: DENV-1, DENV-2, and DENV-3. [6]

While many people infected with DENV may not exhibit symptoms or may only experience mild illness, the virus can also lead to severe cases and even death. Approximately 5% of individuals who acquire dengue fever are likely to develop severe dengue, a condition that may result in shock, internal hemorrhaging, and potentially fatal outcomes. The patient’s clinical profile, infection with a different serotype in the past, infants and pregnant women are at higher risk for developing severe dengue. There are two vaccines, Dengvaxia^®^ and TAK003^®^, indicated for people who inhabit areas with a high circulation of the virus, but specific and effective treatments are not available; therefore, control measures focus on reducing the circulation of the vector [7], and care management relies on the use of analgesics to control symptoms, avoiding non-steroidal anti-inflammatory drugs [4].

Natural products have a prominent role in the drug discovery process. Over 50% of the currently available drugs were initially obtained from natural sources or their design was inspired by nature [8]. Most of the natural compounds found in species of the Asteraceae family that have been reported to exhibit activity against DENV are phenolic compounds. Several flavonoids have demonstrated anti-viral activity against DENV in vitro, including quercetin, quercitrin, kaempferol-3-O-rutinoside, and epicatechin, among others [9,10,11,12]. Various flavonoids widely distributed in the Asteraceae family have demonstrated potential as inhibitors towards DENV proteases such as the NS2b/NS3pro complex in several in silico studies involving molecular docking, including quercetin, rutin, myricetin, luteolin, hesperidin, and others [9]. However, in silico studies could not prove that these compounds could inhibit the virus as a whole. On the other hand, limited research has been conducted on anti-DENV activity of terpenoids. Despite compounds like genudin, andrographolide, stachyonic acid A, ganodermanontriol, and lucidumol A having been demonstrated to be active against DENV both in vitro and in silico [9,13], studies related to the anti-DENV activity of sesquiterpene lactones have not been produced.

In light of this, the aim of this paper was to perform an in vitro screening of plant extracts, pure phenolic compounds, and sesquiterpene lactones isolated from different species of Argentinean Asteraceae in the search for bioactive molecules that could be useful in the drug discovery process against DENV, whose infection has become a relevant public health issue.

## 2. Results

### 2.1. Organic Extracts Prepared from Asteraceae Species

Methanolic and dichloromethane extracts from six Asteraceae species were prepared for in vitro testing against DENV. The selected species, the abbreviations, and the resulting yields (g of extract/100 g plant) of the prepared extracts are shown in Table 1.

### 2.2. Pure Phytochemicals Isolated from Asteraceae

**Figure 1:**
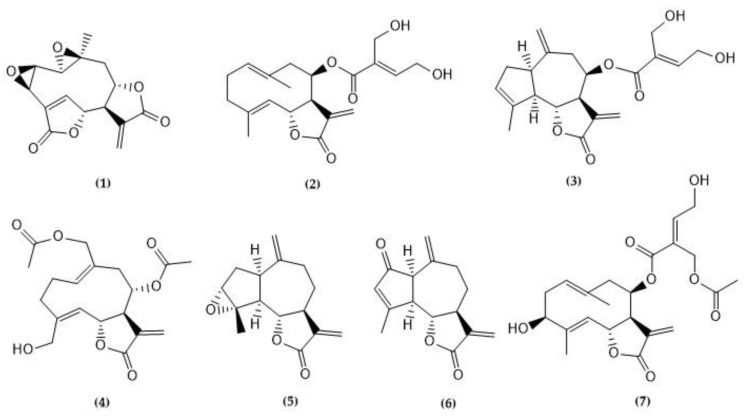
Sesquiterpene lactones isolated from species belonging to the Asteraceae family: Mikanolide (**1**); eupatoriopicrin (**2**); eupahakonenin B (**3**); minimolide (**4**); estafietin (**5**); 2-oxo-8-deoxy-ligustrin (**6**); santhemoidin C (**7**).

**Figure 2:**
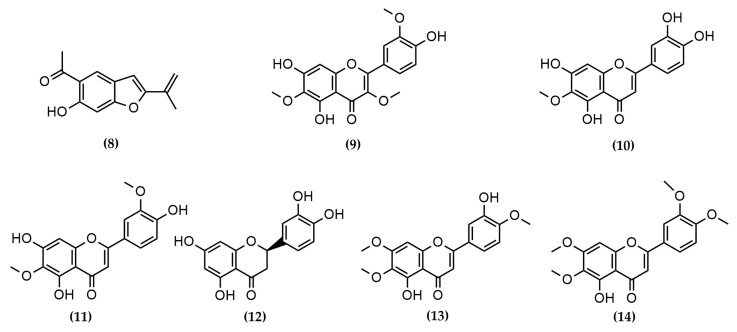
Phenolic compounds isolated from species belonging to the Asteraceae family: Euparin (**8**); jaceidin (**9**); nepetin (**10**); jaceosidin (**11**); eryodictiol (**12**); eupatorin (**13**); 5-demethylsinensetin (**14**).

A total of fourteen phytochemicals, including seven sesquiterpene lactones and seven phenolic compounds, were isolated and purified from Asteraceae species and tested for their antiviral properties in vitro. According to their skeletal arrangement, STLs **1**, **2**, **4**, and **7** correspond to germacranolides, whilst compounds **3**, **5**, and **6** belong to the guaianolide group. The 2D chemical structures of the compounds are shown in Figure 1 and Figure 2.

The phenolic compounds involve the benzofuran euparin (**8**), the flavanone eryiodictiol (**12**), and five flavones (compounds **9**, **10**, **11**, **13**, and **14**).

The identification of the isolated compounds was performed via spectroscopic methods comparing the experimental data with spectra found in the literature. In particular, the ^1^H-NMR and ^13^C-NMR signals of compounds **1**, **2**, **3**, and **6** are specified in this section. The spectra, including ^1^H-^1^H COSY, HSQC, and HMBC of the aforementioned compounds, can be found in the Supplementary Materials of this manuscript (Figures S1–S31).

Mikanolide (**1**): white crystals. IR and MS identical to spectra reported in Herz et al. [14]. ^1^H-RMN [500 MHz, CDCl_3_ + MeOD (9:1)]: δ (ppm) J (Hz) 7.26 (1H, d, 1.5, H-5), 6.47 (1H, dd, 3.5, 1, H-13b), 6.01 (1H, dd, 3, 1, H-13a), 5.29 (1H, dd, 1.5, 3.5, H-6), 4.47 (1H, ddd, 5, 8, 11, H-8), 4.03 (1H, d, 3.5, H-3), 3.38 (1H, dd, 1.5, 3.5, H-2), 3.24 (1H, dddd, 3.5, 4.5, 3.5, 8, H-7), 2.82 (1H, s, H-1), 2.20 (1H, dd, 4.5, 14, H-9a), 2.10 (1H, dd, 11, 14, H-9b), 1.15 (3H, s, H-14). ^13^C-RMN [500 MHz, CDCl_3_ + MeOD (9:1)]: δ (ppm) (carbon type, assignment) 170.24 (C, C-15), 167.64 (C, C-12), 147.46 (C, C-5), 136.68 (C, C-11), 131.05 (C, C-4), 124.46 (CH_2_, C-13), 83.02 (CH, C-6), 77.07 (CH, C-8), 57.99 (CH, C-1), 57.20 (C, C-10), 55.48 (CH, C-2), 50.55 (CH, C-7), 50.27 (CH, C-3), 43.36 (CH_2_, C-9), 21.43 (CH_3_, C-14).

Eupatoriopicrin (**2**): Yellow crystals. IR (ATR), γ_máx_ (cm^−1^): 1738, 1714 (C=O), 1660 (C=C), 3419 (-OH). MS (70 eV) *m*/*z* (int. rel.): 363 [M + H^+^] (0.4%), 362 [M^+^], 231 (11.72%), 216 (3.9%), 176 (9.54%), 131 (13.77%), 113 (4.04%), 119 (40.89%), 105 (27.25%), 97 (52.27%), 95 (100%), 91 (35.3%), 69 (56.88%), 55 (37.74%), 43 (51.56%), 41 (55.06%). ^1^H-RMN (600 MHz, CDCl_3_): δ (ppm) 6.89 (t, *J* = 5.8 Hz, 1H, H-3′), 6.32 (d, *J* = 3.5 Hz, 1H, H-13b), 5.85 (dd, *J* = 6.3, 1.8 Hz, 1H, H-8), 5.63 (d, *J* = 3.0 Hz, 1H, H-13a), 5.20 (m, 1H, H-6), 4.92 (dd, *J* = 11.3, 3.5 Hz, 1H, H-1), 4.81 (d, *J* = 9.9 Hz, 1H, H-5), 4.47 (d, *J* = 5.8 Hz, 2H, H-4′), 4.39 (s, 2H, H-5′), 2.98 (m, 1H, H-7), 2.89 (dd, *J* = 14.5, 4.8 Hz, 1H, H-9a), 2.42 (dd, *J* = 5.2, 2.2 Hz, 1H, H-2b), 2.39 (dd, *J* = 14.5, 2.4 Hz, 1H, H-9b), 2.34 (dd, *J* = 12.1, 5.0 Hz, 1H, H-2a), 2.26 (dd, *J* = 18.0, 5.2 Hz, 1H, H-3b), 2.12 (ddd, *J* = 15.8, 10.7, 5.5 Hz, 1H, H-3a), 1.80 (s, 3H, H-15), 1.50 (s, 3H, H-14). ^13^C-RMN (150 MHz, CDCl_3_): δ (ppm) (carbon type, assignment) 169.57 (C, C-12), 165.77 (C, C-1′), 144.02 (CH, C-3′), 142.57 (C, C-4), 136.63 (C, C-11), 132.93 (C, C-10), 131.93 (C, C-2′), 130.93 (CH, C-5), 127.26 (CH, C-1), 121.21 (CH_2_, C-13), 75.64 (CH, C-6), 72.51 (CH, C-8), 59.22 (CH_2_, C-5′), 57.41 (CH_2_, C-4′), 52.79 (CH, C-7), 44.02 (CH_2_, C-9), 39.43 (CH_2_, C-3), 26.20 (CH_2_, C-2), 19.04 (CH_3_, C-14), 17.49 (CH_3_, C-15).

Eupahakonenin B (**3**): Gum. IR and UV identical to spectra reported in Ito et al. [15]. MS (70 eV) *m*/*z* (int. rel.): 360 [M]^+^ (0.73%), 342 (1.76%), 246 (4.62%), 228 (56.82%), 183 (48.74%), 97 (86.51%), 95 (100%), 69 (77.15%), 41 (51.48%). ^1^H-RMN (600 MHz, CDCl_3_): δ (ppm) 6.87 (t, *J* = 5.8 Hz, 1H, H-3′), 6.32 (d, *J* = 3.5 Hz, 1H, H-13b),5.68 (td, *J* = 5.0, 2.8 Hz, 1H, H-8), 5.60 (s, 1H, H-3), 5.58 (d, *J* = 3.1 Hz, 1H, H-13a), 5.05 (s, 1H, H-14b), 4.92 (s, 1H, H-14a), 4.53 (dd, *J* = 10.4, 8.9 Hz, 1H, H-6), 4.46 (d, *J* = 5.9 Hz, 2H, H-4′), 4.36 (s, 2H, H-5′), 3.22 (m, 1H, H-7), 2.88 (t, *J* = 9.4 Hz, 1H, H-5), 2.60 (t, *J* = 5.7 Hz, 1H, H-9), 2.47 (dd, *J* = 4.9, 2.2 Hz, 1H, H-2), 1.91 (s, 3H, C-15). ^13^C-RMN (150 MHz, CDCl_3_): δ (ppm)(carbon type, assignment) 169.47 (C, C-12), 166.00 (C, C-1′), 143.80 (CH, C-3′), 143.41 (C, C-10), 139.80 (C, C-4), 134.67 (C, C-11), 131.40 (C, C-2′), 126.83 (CH, C-3), 122.14 (CH_2_, C-13), 116.65 (CH_2_, C-14), 79.95 (CH, C-6), 68.30 (CH, C-8), 59.27 (CH_2_, C-4′), 57.43 (CH_2_, C-5′), 56.47 (CH, C-5), 48.59 (CH, C-7), 47.35 (CH, C-1), 40.56 (CH_2_, C-9), 37.43 (CH_2_, C-2), 16.77 (CH_3_, C-15).

2-oxo-8-deoxyligustrin (**6**): White crystals. IR and MS identical to spectra reported in Zdero et al. [16]. ^1^H-NMR (600 MHz, CDCl_3_): δ (ppm) 6.30 (d, *J* = 3.5 Hz, 1H, H-13b), 6.17–6.12 (m, 1H, H-3), 5.58 (d, *J* = 3.1 Hz, 1H, H-13a), 5.12 (s, 1H, H-14b), 4.85 (d, *J* = 0.8 Hz, 1H, H-14a), 4.13 (dd, *J* = 10.6, 8.8 Hz, 1H, H-6), 3.33 (d, *J* = 7.4 Hz, 1H, H-1), 3.23–3.16 (m, 1H, H-5), 2.92 (td, *J* = 12.1, 3.5 Hz, 1H, H-7), 2.54 (dt, *J* = 13.2, 4.9 Hz, 1H, H-9b), 2.34 (t, *J* = 1.2 Hz, 3H, H-15), 2.33–2.27 (m, 1H, H-8b), 2.19 (ddd, *J* = 13.2, 10.6, 5.3 Hz, 1H, H-9a), 1.54–1.43 (m, 1H, H-8a). ^13^C-NMR (151 MHz, CDCl_3_): δ (ppm)(carbon type, assignment) 206.58 (C, C-2), 177.82 (C, C-4), 169.49 (C, C-12), 144.12 (C, C-10), 138.31 (C, C-11), 132.59 (CH, C-3), 121.28 (CH_2_, C-13), 117.28 (CH_2_, C-14), 83.29 (CH, C-6), 56.08 (C, C-1), 53.15 (CH, C-5), 46.09 (CH, C-7), 36.49 (CH_2_, C-9), 31.14 (CH_2_, C-8), 19.94 (CH_3_, C-15).

### 2.3. Cytotoxicity and Antiviral Activity of Extracts from Asteraceae Species 

**Figure 3:**
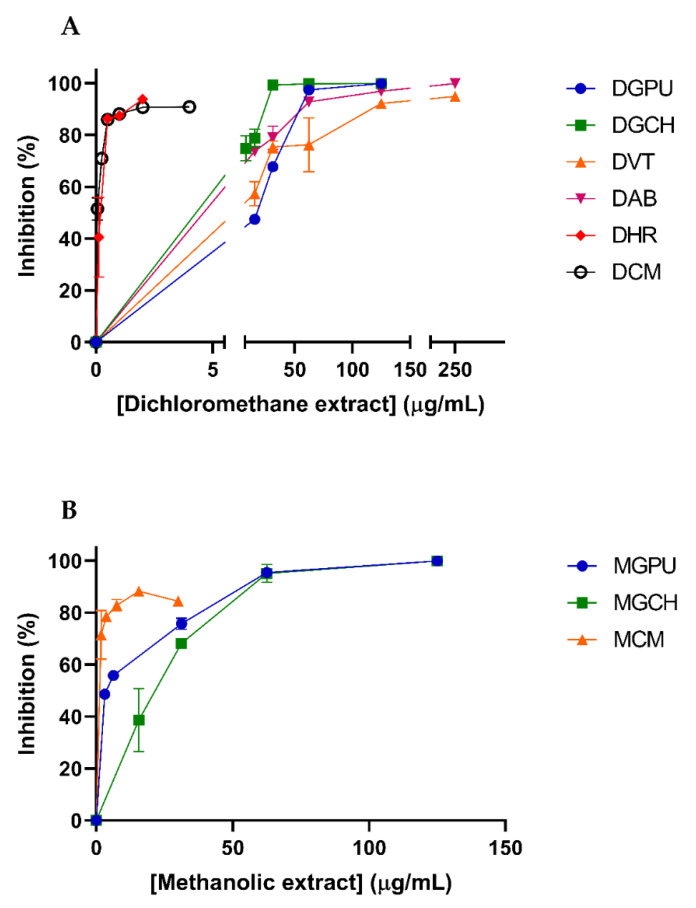
Antiviral activity of dichloromethane (**A**) and methanolic extracts (**B**) and against DENV-2 in Vero cells. Cells were infected with DENV-2 (m.o.i. 0.1 PFU/cell) and, after 1 h adsorption at 37 °C, were treated with medium containing different dilutions of the dichloromethane extracts or vehicle. After 48h of infection, viral yield was determined via plaque assay. Extracts that were considered inactive were not included. Results are expressed as the mean values ± standard deviation (SD).

The dichloromethane and methanolic extracts of the Argentinean Asteraceae species were evaluated against DENV-2. Each set of extracts was first assayed for cytotoxicity on Vero cells. Antiviral activity was afterward evaluated at concentrations below the CC_50_ for each extract (Figure 3).

The dichloromethane extracts were able to inhibit the replication of DENV-2 on Vero cells with EC_50_ values in the range of 53.96–0.11 µg/mL. DCM and DHR showed the lowest EC_50_ values: 0.15 and 0.11µg/mL, respectively. DCM was the most selective extract (SI = 171.10), and DHR showed the second-highest selectivity index (SI = 18.80) (Table 2).

On the other hand, three methanolic extracts, MGPU, MGCH, and MCM, showed inhibition of DENV-2 with EC_50_ values of 3.85, 29.96, and 1.80 µg/mL, respectively (Table 3). From this set of extracts tested, MGPU stands out, since it afforded a high selectivity against DENV-2 (SI > 64.93), followed by MCM with an SI of >17.36.

### 2.4. Cytotoxicity and Ant-Iviral Activity of the Isolated Phytochemicals

**Figure 4:**
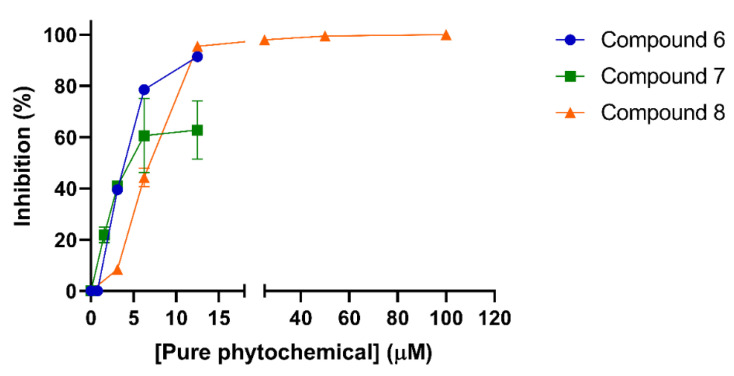
Antiviral activity of pure compounds against DENV-2 in Vero cells. Cells were infected with DENV-2 (m.o.i. 0.1 PFU/cell) and, after 1 h adsorption at 37 °C, were treated with medium containing different dilutions of the pure compounds or vehicle (dimethyl sulfoxide). After 48 h of infection, viral yield was determined via plaque assay. Results are expressed as the mean values ± standard deviation (SD).

Before the evaluation of the anti-DENV-2 activity, the pure natural compounds were submitted to cytotoxicity assays. Antiviral activity was afterward evaluated at concentrations below the CC_50_ for each compound (Figure 4). Most of the compounds tested were inactive in the antiviral assays (Table 4). Two sesquiterpene lactones, 2-oxo-8-deoxy-ligustrin and santhemoidin C (compounds **6** and **7**), exhibited inhibitory effects on DENV-2 with EC_50_s of 3.72 and 3.12 µM and SI values of 5.86 and 6.74, respectively, which would indicate a specific inhibitory action of the compound against virus multiplication at concentrations lower than the CC_50_ (Table 4). Among phenolic compounds, only euparin (compound **8**) showed significant antiviral activity, with an EC_50_ value of 6.81 and high SI (SI > 73.4) (Table 4).

### 2.5. Chromatographic Analysis of the Active Extracts against DENV-2

**Figure 5:**
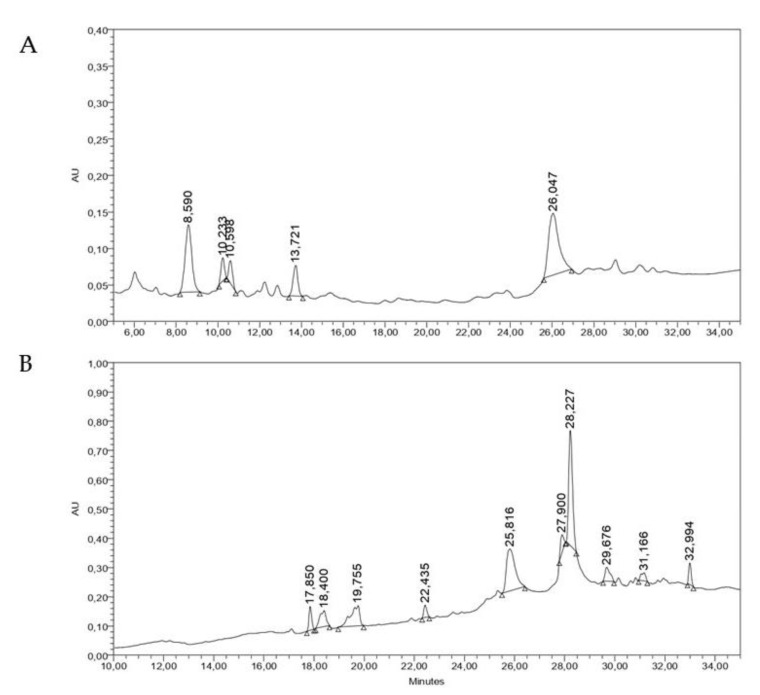
HPLC analysis of (**A**) MGPU and (**B**) MCM. (Chromatographic system: stationary phase: C-18; mobile phase: 15–85% MEOH in 35 min; detector: PDA, 210 nm).

**Figure 6:**
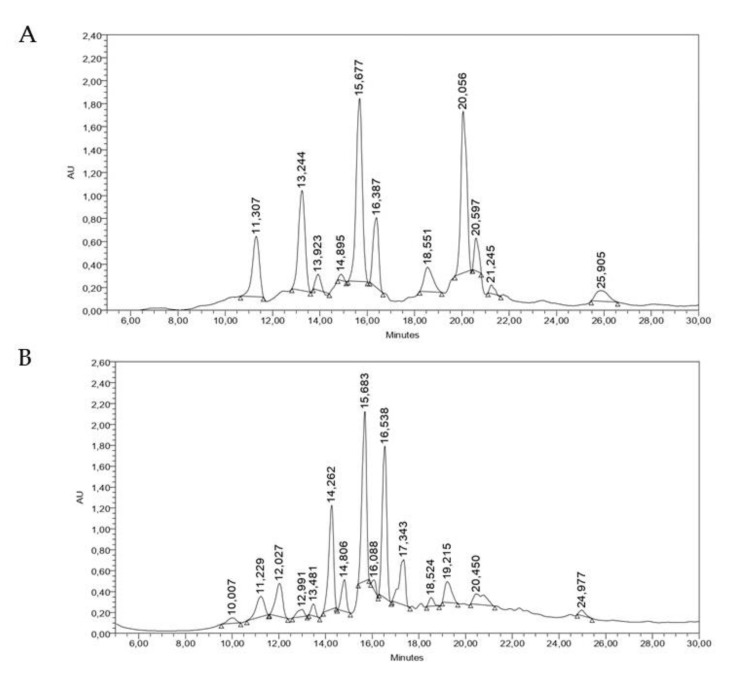
HPLC analysis of (**A**) DHR and (**B**) DCM. (Chromatographic system: stationary phase: C-18; mobile phase: 15–85% MEOH in 35 min; detector: PDA, 210 nm).

Two dichloromethane extracts (DCM and DHR) and two methanolic extracts (MGPU and MCM) were found to be active against DENV-2 and were therefore selected for analysis of their chromatographic behavior via thin-layer chromatography (TLC) and high-performance liquid chromatography (HPLC). The chromatograms obtained from the analysis can be found in Figure 5, Figure 6 and Figure S32. The TLC analysis of MGPU showed one gray band with a retention factor (Rf) of 0.20, while no bands were observed after spraying the plate with the NPR reagent. MCM showed one gray band (Rf = 0.20) and one pink compound (Rf = 0.90). This extract did not show components that developed with NPR. On the other hand, the dichloromethane extracts proved to be constituted of several compounds. DHR showed four major bands (Rf = 0.10, 0.15, 0.80, and 0.90) colored violet, yellow, pink, and pink, respectively, after spraying the plate with *Anisaldehyde sulfuric acid* reagent. The analysis of the extract DCM showed five main compounds (Rf = 0.20, 0.30, 0,80, 0.87, 0.90); the first two compounds appeared as brown bands, while the rest developed as pink bands after spraying with *Anisaldehyde sulfuric acid* reagent (Figure S32).

Regarding the HPLC analysis of the methanolic extracts, two major peaks appeared in the MGPU extract with retention times (RT) of 8.590 and 26.047 min. MCM showed one major peak (RT: 28.227 min) and several minor components (Figure 5). In the UV spectra of these peaks, bands I and II, typically observed in flavonoid compounds, were present (Figure 5).

As was evidenced in the TLC of the extracts, the HPLC analysis proved that the dichloromethane extracts of DHR and DCM were enriched in at least a dozen phytochemicals each. DHR showed two major compounds (RTs: 15.677 and 20.056 min), whilst one major compound with an RT of 15.683 min was observed in the DCM extract. Most of the peaks showed UV spectra with maximums at 210–220 nm (Figure 6).

## 3. Discussion

In this paper, an in vitro screening of the anti-DENV activity of six plant extracts from the Asteraceae family was performed. Results showed that the most active and selective extracts against DENV-2 were DCM, MCM, MGPU, and DHR. This information is indicative that components of these extracts, either isolated or combined, might exhibit antiviral activity. The chromatographic analysis of these extracts via TLC and HPLC showed that the methanolic extracts are composed of a few compounds that would be flavonoids. On the other hand, the dichloromethane extracts presented lipophilic constituents that, according to their chromatographic profiles, UV spectra, and behavior on TLC, are most likely terpenoid compounds.

The dichloromethane extract of *C. macrocephalum* (DCM) was the most active and selective extract on DENV-2. This extract demonstrated an EC_50_ of 0.11 µg/mL with an SI of 171.1, indicating a probably specific mode of action. On the other hand, the methanolic extract of this species (MCM) was also active and selective against DENV-2 but in a smaller magnitude than the DCM. This species has been used as part of Paraguayan folk medicine for anti-inflammatory, sedative, and cardioprotective purposes [17]. Indigenous populations in Argentina have also reported the use of this plant as a curative of coughs and against hepatic affections [18]. The anti-inflammatory properties attributed to this Asteraceae may alleviate several symptoms associated with DENV infection. Furthermore, this extract has been reported to reduce edema and inflammation signs when tested in the TPA-induced ear edema and carrageenan-induced edema assays [19]. Regarding the phytochemical composition of *C. macrocephalum*, steroids and triterpenes such as lupeol, α-amyrin, β-amyrin, and their respective acetates, along with pseudotaraxasterol, have been isolated from its aerial parts. The sesquiterpene lactones macrocephalides A, B, and C have also been reported in this species. In this sense, macrocephalides A and B were active against human cancer cell lines with 50% cell growth inhibitions of 0.576 and 6.37 µM, respectively [20]. Additionally, phenolic compounds were found in extracts of *C. macrocephalum* including the flavonoid taxifolin and the heteroside quercetin-3-O-α-L-rhamnoptranoside-7-O-β-D-glucopyranoside [20]. The anti-inflammatory activity of triterpenes lupeol, lupeol acetate, taraxasterol, and α- and β-amyrin and the flavonoid taxifolin has been reported [17,21]. Since this is the first report of the anti-DENV activity of *C. macrocephalum*, the dichloromethane extract and its constituents could be novel antiviral candidates.

Another extract tested in this work against DENV-2 was the methanolic extract prepared from the aerial parts of *G. pulchella* (MGPU), which demonstrated activity and selectivity of action against this RNA virus (EC_50_ = 3.85 µg/mL; SI > 64.93). The leaves and stems of this species of Asteraceae have been used since the 17th century as a medicinal native plant by an Argentinian indigenous population called “The comechingones” as an anti-poison, febrifuge, and against rheumatic pains [18]. These popular uses could be related to the treatment of high fevers and rheumatic pains frequently associated with dengue fever. The diterpenoid grindelic acid has been isolated and purified from its aerial parts [22]. This diterpene has demonstrated anti-inflammatory properties in vitro, with an inhibitory effect on IL-8, IL-1β, and TNF-α release from LPS-stimulated neutrophils [23]. Most of the phytochemical constituents of *G. pulchella* remain to be studied since there are only a few reports available concerning the chemistry of this species. Given the anti-DENV-2 properties that MGPU demonstrated in this paper, the study of the phytochemistry of *G. pulchella* holds great promise for future research in the search for new antiviral compounds.

The dichloromethane extract of *H. radiatum* (DHR) also showed promising anti-DENV-2 activity (EC_50_ = 0.15 µg/mL; SI = 18.80). This endemic species from South America has not been associated with popular medicinal uses, but its colorful flowers are used for ornamental purposes by locals. The aerial parts of this species have been studied for their chemical composition, affording the flavones jaceosidin and nepetin and the sesquiterpene lactones 9-O-desacetylspathulin-2-O-angelate and 2-desoxypleniradin-4-O-α-L-rhamnopyranoside [24]. Since we tested jaceosidin and nepetin against DENV-2, and these compounds resulted inactive against the virus, the antiviral properties of this Asteraceae species may be due to its sesquiterpene lactones or other unknown constituents, which require further studies into their potential as new anti-DENV molecules.

Concerning the pure phytochemicals isolated from the Asteraceae species tested, the phenolic compound euparin (**8**), and the sesquiterpene lactones 2-oxo-8-deoxyligustrin and santhemoidin C (compounds **6** and **7,** respectively) exerted the strongest anti-DENV-2 activity.

Regarding the sesquiterpene lactones, to our knowledge, we report herein the first anti-DENV properties of compounds of this type. The fact that the other compounds from this group of phytochemicals were inactive against the virus could suggest that a specific mechanism of viral inhibition is involved in the results obtained for these two sesquiterpenes. The moderate selectivity of action of these natural products makes them interesting candidates for the optimization and modification of their chemical structures via semi-synthesis in the search for more active and selective lead molecules in the development of new anti-DENV treatments.

Euparin is an anti-oxidant benzofuran that has been isolated from several species of Asteraceae, and it was the only phenolic compound from the series that showed activity against DENV-2 with selectivity of action. This natural compound has been evaluated for its activity against poliovirus, demonstrating an inhibition in the early stages of the poliovirus replication cycle, from virus adsorption to cells up to the first twenty minutes after infection, probably affecting the penetration and/or the uncoating process [25]. Moreover, this benzofuran exhibited activity against other RNA viruses such as respiratory syncytial virus (RSV) and pseudorabies virus strain RC/79 [26]. On the other hand, euparin derivatives showed protein tyrosine phosphatase 1B (PTP1B) inhibition in vitro [27]. It has been demonstrated that inhibitors of PTP1B can decrease DENV multiplication in HepG2 cells [28]. We can hypothesize that euparin could produce similar effects on DENV-2, and consequently, additional efforts should be invested in the elucidation of its molecular mechanism and the anti-DENV potential of this molecule.

The results obtained in this investigation demonstrate the potential of the Asteraceae family as a prolific source of bioactive phytochemicals. Moreover, the diverse flora and endemic plant species found in South America offer a wealth of raw materials for pharmacological research and investigation.

## 4. Materials and Methods

### 4.1. Plant Materials

The aerial parts of several species of the Asteraceae family were collected from different locations within Argentine territory.

#### 4.1.1. Asteraceae Plant Materials Used to Prepare the Organic Extracts Tested against DENV-2

*Acmella bellidioides* (Sm.) R.K. Jansen (BAF 736) was collected in Entre Rios Province, Concordia Department, Rivadavia Park, near the river Uruguay, in November 2011. *Viguiera tuberosa* Griseb. (BAF 731) was collected in Entre Ríos Province, Uruguay Department, on the road from Concepción del Uruguay to Banco Pelay, in November 2011. The aerial parts of *Campuloclinium macrocephalum* (Less.) DC. (BAF 802) and *Grindelia pulchella* Dunal (BAF 14869) were collected in Entre Ríos Province, Islas del Ibicuy Department: Médanos, national route N12, in December 2012 and March 2019, respectively. *Helenium radiatum* (Less) Seckt (BAF 751) was collected in Buenos Aires Province, Saavedra District, Cura Malal mountains, Abra Agua Blanca, in February 2012. Lastly, aerial parts of *Grindelia chiloensis* (Cornel.) Cabrera (BAF 16000) were collected in Neuquén Province, Collon Cura Department, between Chocon and Piedra del Aguila, in January 2019.

The plant materials were harvested conservatively to preserve the genetic resource (10–15% of aerial parts of each plant were cut using scissors). Voucher specimens are available at the Museo de Farmacobotanica, Facultad de Farmacia y Bioquímica, Universidad de Buenos Aires, Argentina. The identification of the materials was made by the renowned taxonomists Dr. Gustavo Giberti and Hernan Bach.

#### 4.1.2. Asteraceae Plant Materials Used to Obtain the Pure Phytochemicals Tested against DEN-2

The aerial parts of *Stevia satureiifolia* (Lam.) Sch. Bip. var. *satureiifolia* (BAF 744) were collected in Buenos Aires Province, near Tandil, Animas mountains, in February 2012. *Stevia aristata* D. Don ex Hook. and Arn. (BAF 797) was collected in Entre Ríos Province, La Paz Department, national route N1, in December 2012 [29]. *Stevia maimarensis* (Hieron.) Cabrera (BAF 12264) was collected in the Jujuy Province, Tilcara Department: Perchel, in March 2017. *Stevia alpina* Griseb. (BAF 12266) was collected in Catamarca Province, Provincial route 307, Km 49, in April 2015, while *Stevia gilliesii* Hook. and Arn. (BAF 12267) was collected in Catamarca Province, Paclín Department, Provincial route N38, in March 2015 [30]. *Mikania minima* (Baker) B.L. Rob. (BAF 12268) aerial parts were collected in Tucumán Province in April 2015 [31]. *Urolepis hecatantha* (DC.) R. King and H. Robins (BAF 16100) was collected in Buenos Aires Province, Provincial Route 40 near Norberto de la Riestra, in March 2018 [32]. Voucher specimens are available at the Museo de Farmacobotanica, Facultad de Farmacia y Bioquímica, Universidad de Buenos Aires, Argentina.

The plant material of the species *Mikania micrantha* Kunth (LIL609699), was harvested in Tucumán Province, Trancas Department, near Vipos river, in June 2009 [30]. A voucher specimen is deposited at the Instituto Miguel Lillo, Facultad de Ciencias Naturales, Universidad Nacional de Tucumán [31].

From the species *Chromolaena arnottiana* (Griseb.) R.M. King and H. Rob. (J. de D. Muñoz 3535), the aerial parts used in this paper were collected in Entre Ríos Province, Paraná, Oro Verde, in March 2004. A voucher specimen can be found at the Herbarium of the Facultad de Ciencias Agropecuarias de Entre Ríos, Argentina [33].

### 4.2. Plant Extracts

#### 4.2.1. Plant Extracts Obtention

Once air-dried, the aerial parts of the Asteraceae species mentioned in Section 4.1.1 were extracted via maceration at room temperature with either dichloromethane (CH_2_Cl_2_) or methanol (MEOH). Dichloromethane extracts were prepared at a concentration of 10% *w*/*v* for an extraction period of 5 min followed by filtration (Schleicher and Schuell—Whatman, grade 0859, medium, smooth, 90 mm) [34]. This process was repeated twice for each plant material, and the resulting extracts were combined and taken to dryness under vacuum in a rotary evaporator. The plant material was then extracted with MEOH for 24 h under the same conditions. The extracts were unified, filtered, and taken to dryness under reduced pressure.

#### 4.2.2. Chromatographic Analysis of the Active Extracts against DENV-2

##### Thin Layer Chromatography Analysis (TLC)

The chromatographic analyses of dichloromethane extracts of *H. radiatum* (DHR) and *C. macrocephalum* (DCM) were performed on Silicagel plates (F_254_, Merck) developed with hexane–ethyl acetate (HX:ETAC) (1:1) and using anisaldehyde sulfuric acid as a spray reagent. Methanolic extracts of *G. pulchella* (MGP) and *C. macrocephalum* (MCM) were assayed on Silicagel plates (F_254_, Merck) developed with ETAC:METOH (10:5) and sprayed with natural products reagent (NPR) and anisaldehyde sulfuric acid reagent. Rf values of the major bands were calculated.

##### High Performance Liquid Chromatography Analysis (HPLC)

The chromatographic HPLC profiles of DHR, DCM, MGP, and MCM were performed using Waters equipment with a photodiode array detector (Waters 2996), a Rheodyne injection valve (20 μL), pump (Waters Delta 600), Waters 600 controller, and in-line degasser. A reversed-phase column (LiChroCART 125-4, 5 µm Supersphere 100 RP-18, Merck, Darmstadt, Germany) was used, and the photodiode array detector was set at 210 nm. The four extracts were dissolved in MEOH:H_2_O (1:1) at a 10 mg/mL concentration. The solutions were filtered with a nylon filter (0.45 µM, Agilent) to be eluted with a gradient of water (A) and MEOH (B) from 15% B to 100% B in 30 min. The flow rate was 1.0 mL/min, and the elution was performed at room temperature. Chromatograms were recorded and processed using the Empower Pro 3 software. The water employed to prepare the mobile phase was of ultrapure quality (Milliq). Acetonitrile (HPLC) J. T. Baker and methanol (HPLC) J. T. Baker were used.

### 4.3. Isolation of Pure Phytochemicals from Asteraceae Species

#### 4.3.1. Previously Reported Compounds from Asteraceae Species

The CH_2_Cl_2_:MEOH (1:1) extract prepared via maceration from the aerial parts of *Mikania micrantha* H. B. K. was fractionated through column chromatography. Next, the active fractions were combined and subjected to open-column chromatography on Silicagel 60 eluted with a gradient of HX, CH_2_Cl_2_, ETAC, and MEOH. From the fractions eluted with CH2Cl2:ETAC (95:5), a crystalline precipitate was obtained to be later purified and identified as mikanolide (compound **1**), as described by Laurella et al. [26].

The compounds eupatoriopicrin (**2**), eupahakonenin B (**3**), minimolide (**4**), and estafietin (**5**) were isolated from the aerial parts of *Stevia maimarensis* (Hieron.) Cabrera, *Stevia gilliesii* Hook. and Arn., *Mikania minima* (Baker) B.L. Rob., and *Stevia alpina* Griseb., respectively, following the protocols outlined by Elso et al. [30]. In this sense, CH_2_Cl_2_ crude extracts were obtained from the aerial parts of each plant species. The resulting *S. maimarensis* and *S. gilliesii* dichloromethane crude extracts were dewaxed and fractionated via Silicagel 60 column chromatography that was isocratically eluted with CH_2_Cl_2_:ETAC (1:2). The sesquiterpene lactone (STL) eupatoriopicrin (**2**) precipitated as yellow crystals from *S. maimarensis* fractions. At the same time, eupahakonenin B (**3**) was obtained as a greenish gum from *S. gilliesi* fractions. On the other hand, *M. minima* and *S. alpina* crude extracts were fractionated via Silicagel column chromatography and eluted isocratically with CH_2_Cl_2_:ETAC (2:1) and (9.5:0.5) to afford the STLs minimolide (**4**) and estafietin (**5**), respectively. Both substances precipitated as white needle-shaped crystals [30].

The STLs 2-oxo-8-deoxyligustrin (**6**) and santhemoidin C (**7**) were extracted and purified from *Stevia alpina* Griseb. and *Stevia satureiifolia* (Lam.) Sch. Bip. var. *satureiifolia* respectively, as reported by de Heluani et al. (1989) [35] and Borgo et al. [29], respectively. The CH_2_Cl_2_ extract of *S. alpina* was fractionated via column chromatography eluting with chloroform–diethyl ether (CHCl_3_:Et_2_O) to afford 2-oxo-8-deoxyligustrin (**6**), which was later purified by HPLC. A CH_2_Cl_2_ extract of *S. satureiifolia* var. *satureiifolia* was prepared and dewaxed as aforementioned to be fractionated via column chromatography on Silicagel 60 eluting with a gradient of CH_2_Cl_2_:ETAC. Crystals of Santhemoidin C (**7**) precipitated from the fractions eluted with CH_2_Cl_2_:ETAC (3:7) [29]. 

The identification of these phytochemicals was performed via spectroscopic methods comparing experimental spectra with literature data [35,36,37,38].

From the dewaxed dichloromethane extract of *Urolepis hecatantha* (DC) R. King and H. Robins, the phenolic compounds euparin (**8**) and jaceidin (**9**) were obtained and later purified as published [32]. Thus, the dewaxed dichloromethane extract of *Urolepis hecatantha* underwent fractionation via silica gel column chromatography, eluted with a gradient of CH_2_Cl_2_:ETAC. Yellow acicular crystals of euparin (**8**) were obtained from fractions eluted with 100% CH_2_Cl_2_ and CH_2_Cl_2_:ETAC 9:1, while jaceidin (**9**) was purified from fractions eluted with CH_2_Cl_2_:ETAC (7:3) and (6:4).

The flavonoids nepetin (**10**), jaceosidin (**11**), and eryodictiol (**12**) were extracted from *Chromolaena arnottiana* (Griseb.) R.M. King and H. Rob., according to the procedures outlined by Clavin et al. [33]. In this context, the powdered aerial parts of *Chromolaena arnottiana* were extracted successively via maceration with CH_2_Cl_2_ and a 50:50 *v/v* ethanol–water solution. The extracts were then filtered and concentrated under a vacuum. The dichloromethane extract was fractionated on CeliteR, followed by column chromatography on Silicagel. Fractions containing the compounds of interest were combined and purified in a Sephadex LH-20 column. From these final fractions, nepetin (**10**) and jaceosidin (**11**) were isolated by HPLC purification [33]. Moreover, the hydroalcoholic extract was suspended in distilled water and extracted in a semicontinuous liquid–liquid extractor successively with Et_2_O and ETAC. The presence of eriodictyol (**12**) and nepetin (**10**) was confirmed in the Et_2_O fraction [33].

These compounds were identified through spectroscopic analysis and by comparison of their spectral data with literature references [39,40,41,42,43,44,45].

#### 4.3.2. Novel Phytochemicals Isolated from Asteraceae Species

The dichloromethane extract of *Stevia satureiifolia* var. *satureiifolia* (Lam.) Sch. Bip. was processed in order to eliminate sterols and lipids. Therefore, 40 g of extract was suspended in 200 mL of ethanol–water (70:30) to be partitioned with hexane (HX) (3 × 60 mL). The hydroalcoholic suspension was later extracted with dichloromethane (CH_2_Cl_2_) (3 × 60 mL), and the resulting CH_2_Cl_2_ solutions were gathered and taken to dryness to obtain the semi-purified and dewaxed CH_2_Cl_2_ extract. This extract (5.0 g) was later fractionated in a Silicagel 60 column (40 × 4.5 cm, 300 g, 230–400 mesh) with a gradient of hexane and ethyl acetate (HX:ETAC). The fractions were tested via thin-layer chromatography (TLC), using Silicagel F_254_ as the stationary phase (SP), HX:ETAC (5:5) as the mobile phase (MP), and sulfuric anisaldehyde as the spraying reagent (SR). In the fractions eluted with HX:ETAC (5:5), one major compound that developed as one yellow band in the TLC assessment (Rf: 0.5) was observed. This compound was identified as the flavonoid eupatorin (**13**).

The crude extract of *Stevia aristata* D. Don ex Hook. and Arn. was dewaxed with the methodology explained above. A total of 4.85 g of this extract was submitted to fractionation in Silicagel 60 column chromatography (40 × 4.5 cm, 300 g, 230–400 mesh) with an elution gradient of hexane–ethyl acetate (HX:ETAC) (100:0–0:100). The fractions were tested via TLC (SP: Silicagel F_254_; MP: HX:ETAC (5:5); and SR: anisaldehyde sulfuric acid). In the fractions eluted with HX:ETAC (6:4) and (5:5), one major compound that appeared as one yellow band in the TLC analysis (Rf: 0.6) was observed. After evaporation of the solvents of the aforementioned fractions, yellow crystals appeared, which were repeatedly washed with ETAC and MEOH. This compound was later identified as the flavonoid 5-demethylsinensetin (**14**).

The spectroscopic data of these compounds can be found in the Supplementary Materials of this paper.

### 4.4. Identification of Pure Compounds from Asteraceae

The identities of the isolated compounds were determined via proton nuclear magnetic resonance (^1^H-NMR) and carbon nuclear magnetic resonance (^13^C-NMR), heteronuclear single quantum correlation (HSQC), heteronuclear multiple bond correlation (HMBC), correlated spectroscopy (COSY) (Bruker Advance 600) (600 MHz in CDCl_3_), electron impact mass spectrometry (EI-MS), and spectrophotometry (UV), comparing experimental spectra with literature data.

### 4.5. Cells and Virus

Vero (African green monkey kidney, ATCC CCL-81) cells were grown in Eagle’s minimum essential medium (MEM, Gibco™ Thermo Fisher Scientific, Waltham, MA, USA) supplemented with 5% heat-inactivated newborn bovine serum (NBS, Gibco™ Thermo Fisher Scientific, Carlsbad, CA, USA) and 1.5 g/L of sodium bicarbonate in a 5% CO_2_ atmosphere. For the maintenance medium (MM), the serum concentration was reduced to 1.5%. The C6/36 HT mosquito cell line from *Aedes albopictus* (ATCC CRL-1660), adapted to grow at 33 °C, was cultured in L-15 Medium (Leibovitz) supplemented with 0.3% tryptose phosphate broth, 0.02% glutamine, 1% MEM non-essential amino acids solution, and 5% FBS. The original stock of DENV-2 strain NGC was prepared in C6/36 cells and titrated via plaque formation in Vero cells.

### 4.6. Cytotoxicity Assay

Confluent cell monolayers in 96-well plates were exposed for 48 h to serial two-fold compound dilutions, four wells for each concentration, and then viability was tested. The concentration range evaluated for extracts was 1–250 µg/mL, and for pure compounds, it was 1–200 µM or 1–100 µM depending on the phytochemical. In all cases, the concentration of the solvent did not exceed 0.01% of dimethyl sulfoxide (DMSO) or methanol. Cell cultures treated with 0.01% concentration of vehicle (DMSO or methanol) were taken as cell control. Cell viability determinations were performed by the 3-(4,5-dimethylthiazol-2-yl)-2,5-diphenyl tetrazolium bromide (MTT, Sigma-Aldrich, St. Louis, MO, USA) method, adding 10 µL of MM containing MTT reagent (final concentration 0.5 mg/mL) to each well. After 2 h of incubation at 37 °C, the supernatants were removed, 100 µL of ethanol was added per well to solubilize the formazan crystals, and absorbance was measured at 595 nm [46]. Viability values (% respect to cell control): The cytotoxic concentration 50% (CC_50_) was calculated via non-linear regression using GraphPad Prism (v9.0.) software as the compound concentration required to reduce the MTT signal by 50% compared to cell controls treated with vehicle (DMSO or methanol), and it is presented as the mean ± standard deviation (SD) obtained from three independent experiments.

### 4.7. Antiviral Activity Assay

Antiviral activity was evaluated with a virus yield inhibition assay. Cells grown in 24-well microplates were infected with DENV-2 at a multiplicity of infection (m.o.i.) of 0.1 PFU/cell, and after 1 h adsorption at 37 °C, the unadsorbed virus was removed by washing with PBS and refed with MM containing different two-fold dilutions of each compound or vehicle (DMSO or methanol). The concentration range used to evaluate the antiviral activity was variable depending on the CC_50_ of the extracts or compounds (DGPU, DGCH, DAB; MGCH: 7.8–125 µg/mL; DVT: 15.6–250 µg/mL; DHR: 0.1–2 µg/mL; DCM: 0.06–4 µg/mL; MGPU, MVT: 1.6–125 µg/mL; MAB: 0.8–12.5 µg/mL; MHR: 3–50 µg/mL; MCM: 1.8–30 µg/mL; 1, 2, 3: 0.1–2 µM; 4: 0.2–3 µM; 5, 7: 1.6–12.5 µM; 6, 14: 0.8–12.5 µM; 8: 3.1–100 µM; 9, 10, 11: 3–50 µM; 12, 13: 1.6–25 µM). The concentration of the solvent used for the control was the maximum present in the highest concentration of the extract or compound evaluated. After 48 h of infection, supernatants were collected and cooled down, and serial dilutions were prepared to infect Vero cell monolayers to measure virus yield via the PFU method. After viral adsorption for 1 h at 37 °C, the virus excess was removed; MM supplemented with 0.7% methylcellulose was added and cells were incubated for 1 week at 37 °C in a 5% CO_2_ incubator. Finally, cell monolayers were fixed with 10% formaldehyde, washed, and stained with a 1% solution of crystal violet, and PFU were counted. Inhibition values (% respect to viral control): The effective concentration 50% (EC_50_), as the concentration required to reduce virus yield by 50% in the compound-treated cultures, was calculated via non-linear regression using GraphPad Prism (v9.0) software, compared with viral control treated with vehicle (DMSO or methanol), and presented as the mean ± SD obtained from three independent experiments.

## 5. Conclusions

Currently, prevention and control of the vector are the measures most employed for the prevention of dengue fever since no specific antiviral chemotherapy is currently available for the treatment of this disease. Nature has been a source of bioactive compounds that could serve as medicinal treatments. In the present study, we identified potent and selective inhibitors of pathogenic DENV-2 after screening dichloromethane and methanolic extracts and phenolic and sesquiterpene lactone compounds from different species of Argentinean Asteraceae. The screening of a variety of dichloromethane and methanolic extracts allowed us to detect extracts with effective inhibitory action exerted during the intracellular multiplication of DENV-2. Euparin, a member of the phenolic compound group, represents an interesting molecule that deserves to be further studied. The elucidation of the mechanism of action of this selective inhibitor of DENV-2, as well as the evaluation of the effect against other dengue serotypes and in vivo assays, should be considered. The determination of the pharmacokinetic profile of this compound should also be recommended as part of the antiviral drug discovery and development process.

## Figures and Tables

**Table 1:** Asteraceae species used to prepare dichloromethane and methanolic extracts and their respective abbreviations and yields.

Species	Dichloromethane Extract		Methanolic Extract	
	Abbreviation	Yield (%)	Abbreviation	Yield (%)
*Acmella bellidioides*	DAB	0.2	MAB	2.7
*Campuloclinium macrocephalum*	DCM	1.6	MCM	8.7
*Grindelia pulchella*	DGPU	11.5	MGPU	13.7
*Grindelia chiloensis*	DGCH	15.5	MGCH	6.8
*Helenium radiatum*	DHR	3.5	MHR	8.1
*Viguiera tuberosa*	DVT	0.3	MVT	8.5

**Table 2:** CC_50_ and IC_50_ values of dichloromethane extracts.

Extract	CC_50_ (µg/mL)	EC_50_ (µg/mL)	SI
DGPU	˃125.00	26.58 ± 4.80	˃4.70
DGCH	266.50 ± 48.10	39.68 ± 1.20	6.72
DVT	˃250.00	53.96 ± 1.39	>4.63
DAB	230.80 ± 55.25	33.15 ± 1.09	6.96
DHR	2.82 ± 1.21	0.15 ± 0.06	18.80
DCM	18.82 ± 1.26	0.11 ± 0.06	171.10

Values are the mean of three determinations ± SD. Cytotoxic concentration 50%: compound concentration required to reduce cell viability by 50%. Effective concentration 50%: compound concentration required to reduce DENV-2 yield by 50%. Selectivity index: ratio CC_50_/EC_50_.

**Table 3:** CC_50_ and IC_50_ values of methanolic extracts.

Extract	CC_50_ (µg/mL)	EC_50_ (µg/mL)	SI
MGPU	˃250.00	3.85 ± 1.28	˃64.93
MGCH	˃250.00	29.96 ± 5.81	˃8.34
MVT	˃31.25	I	-
MAB	˃15.60	I	-
MHR	˃62.50	I	-
MCM	˃31.25	1.80 ± 0.09	˃17.36

Values are the mean of three determinations ± SD. Cytotoxic concentration 50%: compound concentration required to reduce cell viability by 50%. Effective concentration 50%: compound concentration required to reduce DENV-2 yield by 50%. Selectivity index: ratio CC_50_/EC_50_. I: The extract was inactive at tested concentrations.

**Table 4:** CC_50_ and IC_50_ values of pure phytochemicals **1**–**14**.

Compound	CC_50_ (µM)	EC_50_ (µM)	SI
**1**	6.69 ± 0.49	I	-
**2**	10.80 ± 0.88	I	-
**3**	11.15 ± 0.36	I	-
**4**	10.63 ± 0.55	I	-
**5**	23.46 ± 0.99	I	-
**6**	21.81 ± 4.15	3.72 ± 0.28	5.86
**7**	21.04 ± 3.65	3.12 ± 1.39	6.74
**8**	>500	6.81 ± 0.20	>73.4
**9**	>100	I	-
**10**	>100	I	-
**11**	>100	I	-
**12**	>100	I	-
**13**	>200	I	-
**14**	183.70 ± 27.80	I	-

Values are the mean of three determinations ± SD. Cytotoxic concentration 50%: compound concentration required to reduce cell viability by 50%. Effective concentration 50%: compound concentration required to reduce DENV-2 yield by 50%. Selectivity index: ratio CC_50_/EC_50_. I: The compound was inactive at tested concentrations.

## Data Availability

Data are contained within the article and Supplementary Materials.

## Supplementary Materials

The following supporting information can be downloaded at https://www.mdpi.com/article/10.3390/molecules29040814/s1: Figure S1: Chemical structure of mikanolide; Figure S2: ^1^H-NMR spectrum of mikanolide; Figure S3: ^13^C-NMR spectrum of mikanolide; Figure S4: HSQC spectrum of mikanolide; Figure S5: HMBC spectrum of mikanolide; Figure S6. ^1^H-^1^H COSY spectrum of mikanolide; Figure S7: Chemical structure of eupatoriopicrin; Figure S8: ^1^H-NMR spectrum of eupatoriopicrin; Figure S9: ^13^C-NMR spectrum of eupatoriopicrin; Figure S10: HSQC spectrum of eupatoriopicrin; Figure S11: ^1^H-^1^H COSY spectrum of eupatoriopicrin; Figure S12: Chemical structure of eupahakonenin B; Figure S13. ^1^H-NMR spectrum of eupahakonenin B; Figure S14. ^13^C-NMR spectrum of eupahakonenin B; Figure S15. HSQC spectrum of eupahakonenin B; Figure S16: ^1^H-^1^H COSY spectrum of eupahakonenin B; Figure S17: Chemical structure of 2-oxo-8-deoxyligustrin; Figure S18: ^1^H-NMR spectrum of 2-oxo-8-deoxyligustrin; Figure S19: ^13^C-NMR spectrum of 2-oxo-8-deoxyligustrin; Figure S20: HSQC spectrum of 2-oxo-8-deoxyligustrin; Figure S21: HMBC spectrum of 2-oxo-8-deoxyligustrin; Figure S22: ^1^H ^1^H -COSY spectrum of 2-oxo-8-deoxyligustrin; Figure S23: Chemical structure of eupatorin; Figure S24: ^1^H-NMR spectrum of eupatorin; Figure S25: ^13^C-NMR spectrum of eupatorin; Figure S26: Chemical structure of 5-demethylsinensetin; Figure S27: ^1^H-NMR spectrum of 5-demethylsinensetin; Figure S28: ^13^C-NMR spectrum of 5-demethylsinensetin; Figure S29: HSQC spectrum of 5-demethylsinensetin; Figure S30: HMBC spectrum of 5-demethylsinensetin; Figure S31: ^1^H-^1^H COSY spectrum of 5-demethylsinensetin; Figure S32: TLC analysis of methanolic and dichloromethane extracts MGPU, MCM, DHR and DCM.
