# Supplementary material for: Plant Extracts and Phytochemicals from the Asteraceae Family with Antiviral Properties
Source: Molecules. 2024 Feb 9;29(4):814. doi: 10.3390/molecules29040814 (PMC10891539; doi:10.3390/molecules29040814)
Supplement: Supplementary file 1 [file molecules-29-00814-s001.zip › molecules-2802394-supplementary.pdf]

Supplementary Material

Article

Plant extracts and phytochemicals from the Asteraceae family with antiviral properties

Jimena Borgo[1,2,†], Mariel S. Wagner[3,†], Laura C. Laurella[1,2], Orlando G. Elso[2,4], Mariana G. Selener[2], María Clavin[2], Hernán Bach[5], César A.N. Catalán[6], Augusto E. Bivona[7,8], Claudia S. Sepúlveda[9,‡,*], Valeria P. Sülsen[1,2,‡,*]

[1] Instituto de Química y Metabolismo del Fármaco (IQUIMEFA), CONICET-Universidad de Buenos Aires, Junín 956, piso 2, Buenos Aires C1113AAD, Argentina

[2] Facultad de Farmacia y Bioquímica, Universidad de Buenos Aires, Junín 956, piso 2, Buenos Aires C1113AAD, Argentina

[3] Laboratorio de Estrategias Antivirales, Departamento de Química Biológica, Facultad de Ciencias Exactas y Naturales, Universidad de Buenos Aires, Int. Güiraldes 2160, piso 4, Buenos Aires C1428EGA, Argentina.

[4] Unidad de Microanálisis y Métodos Físicos Aplicados a Química Orgánica (UMYMFOR), Facultad de Ciencias Exactas y Naturales, CONICET-Universidad de Buenos Aires, Ciudad Universitaria, Pabellón 2, piso 3, Buenos Aires C1428EGA, Argentina

[5] Instituto Nacional de Tecnología Agropecuaria (INTA) Gobernador Guillermo Udaondo 1695 Estación Experimental Agropecuaria Área Metropolitana de Buenos Aires, EEA AMBA Udaondo Buenos Aires, B1713 AAW, Argentina

[6] Universidad Nacional de Tucumán, Facultad de Bioquímica, Química y Farmacia, Instituto de Química Orgánica, Ayacucho 471, (T4000INI), San Miguel de Tucumán, Tucumán, Argentina

[7] Instituto de Estudios de la Inmunidad Humoral Prof. Ricardo A. Margni (IDEHU), CONICET-Universidad de Buenos Aires, Junín 956, piso 4, Buenos Aires C1113AAD, Argentina

[8] Instituto de Investigaciones en Microbiología y Parasitología Médica (IMPaM), CONICET-Universidad de Buenos Aires, Paraguay 2155, piso 13, Buenos Aires C1121ABG, Argentina

[9] Instituto de Química Biológica de la Facultad de Ciencias Exactas y Naturales (IQUIBICEN) CONICET-Universidad de Buenos Aires, Int. Güiraldes 2160, piso 4, Buenos Aires C1428EGA, Argentina.

† These authors contributed equally to this work.

‡ The work was co-directed by both authors.

* Correspondence: vsulsen@ffyb.uba.ar; Tel.: +54 (011) 5287-4272 and claudia@qb.fcen.uba.ar

1. Chemical structure and spectral data of mikanolide (compound 1)

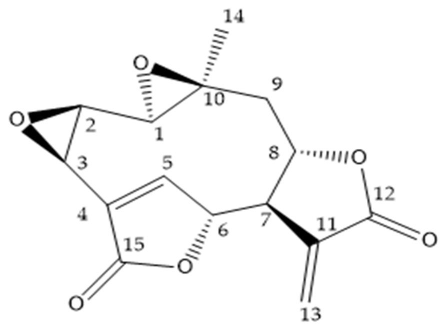

Figure S1. Chemical structure of mikanolide

UV (ʎ máx, nm) MeOH: 207 nm, IR (KBr), γ máx (cm-1): 1764 (C=O), 1640 (C=C)

MS (70 eV) m/z (int. rel.): 261 [M-1-CO^+](2.8%), 243 [M-1-CO-H_2O^+](4.3%), 215 [M-1-CO-H_2O-CO^+](4.9%), 229 [M-H_2O-Me-CO^+](1.2%), 190 (3.1%), 165 (3.9%), 147 (2.9%), 125 (7.8%), 111 (29.1%), 97 (30.5%), 95 (100%), 91 (41.3%), 77 (40.3%), 55 (41.3%), 43 (58.9%).

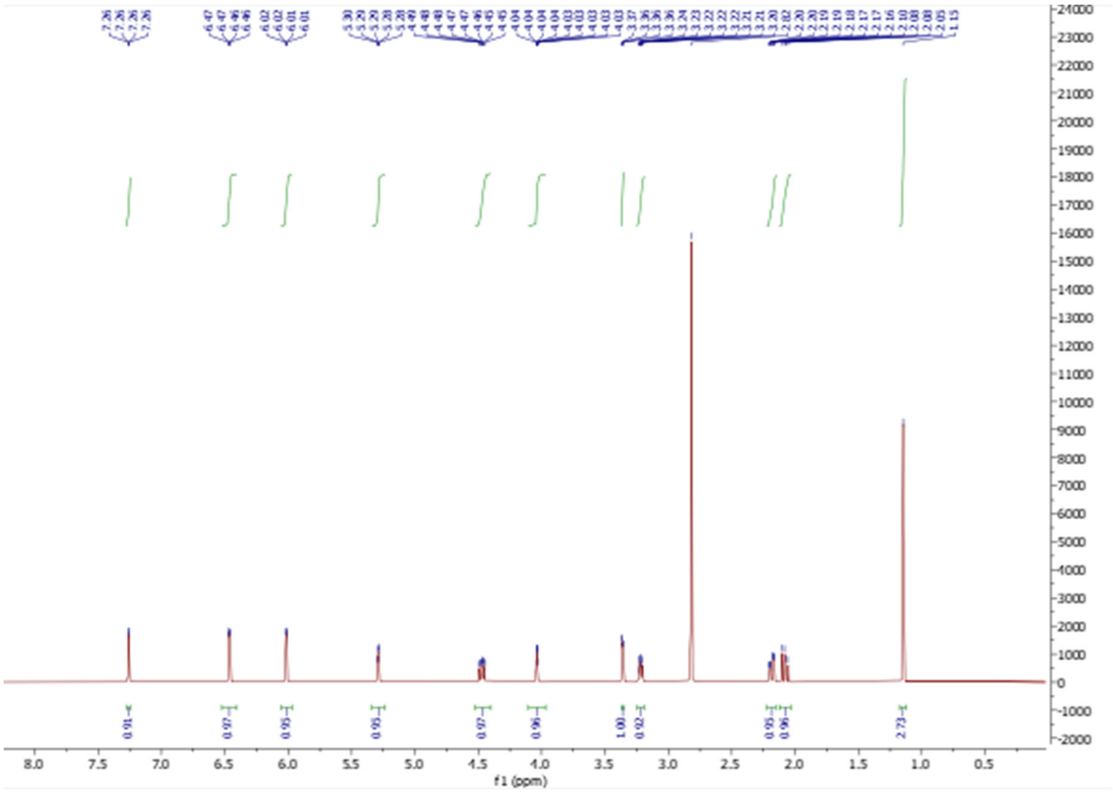

Figure S2. ^1H-NMR spectrum of mikanolide

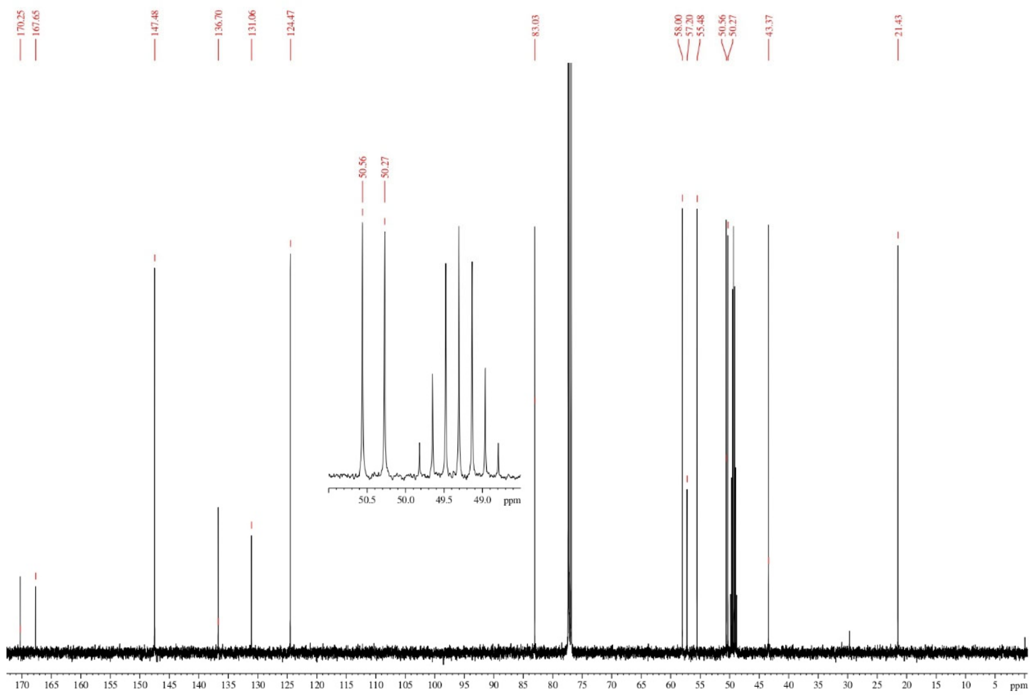

Figure S3. ^{13}C-NMR spectrum of mikanolide

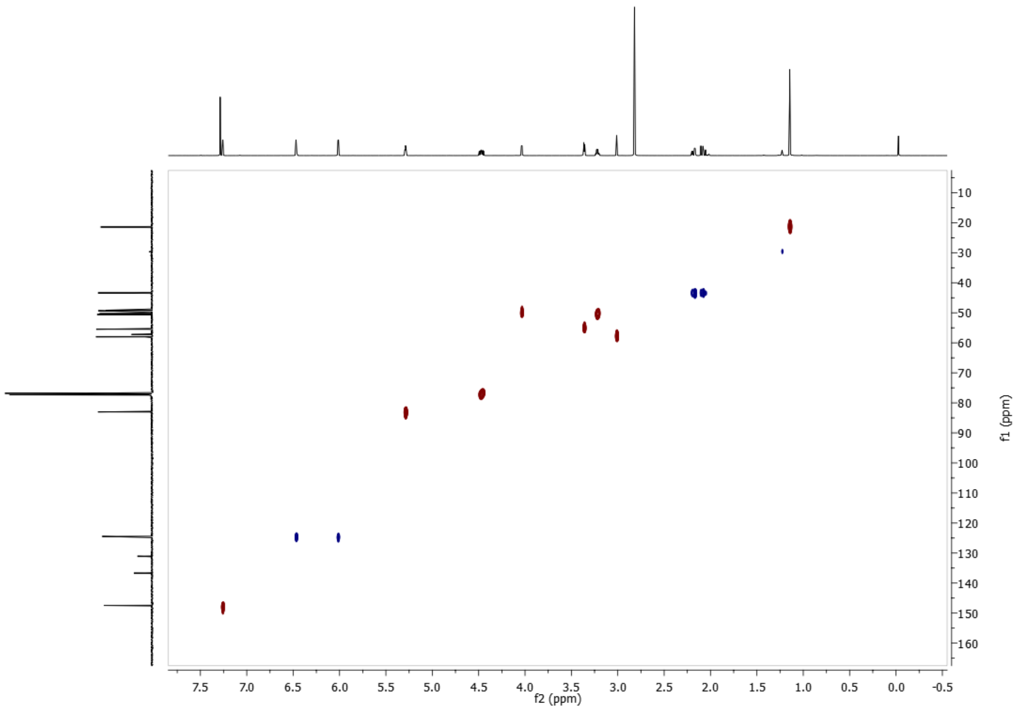

Figure S4. HSQC spectrum of mikanolide

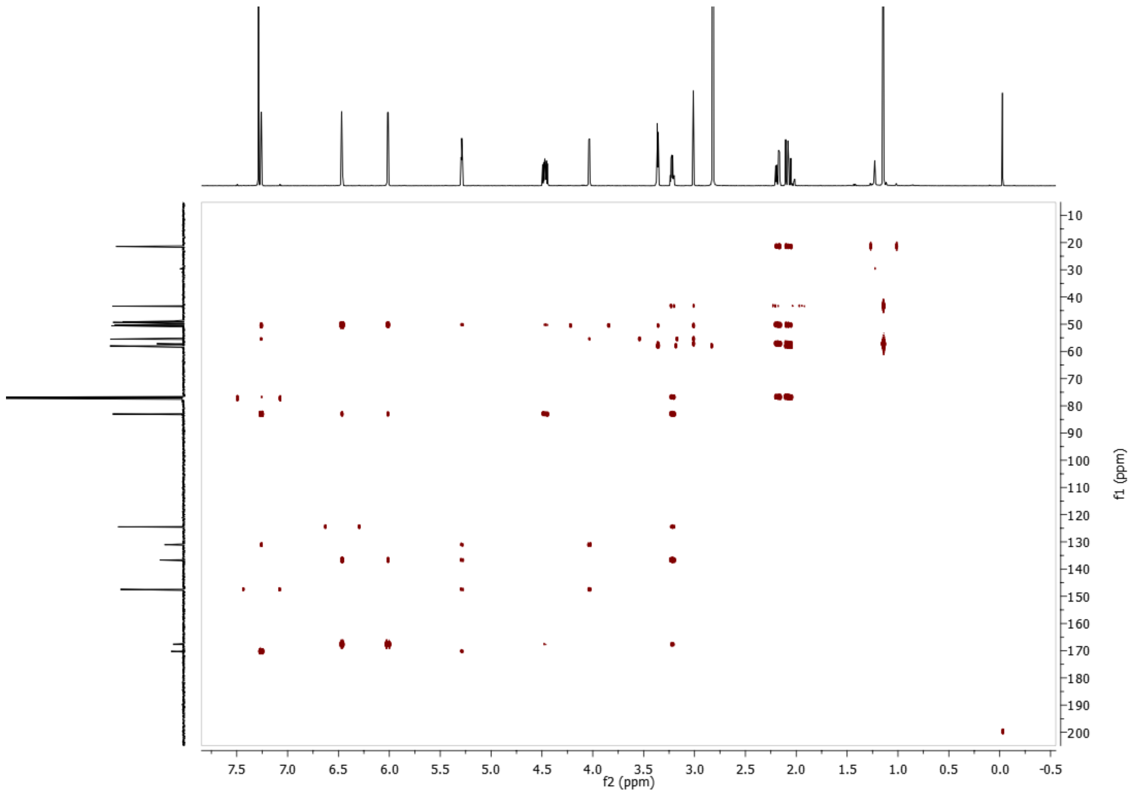

Figure S5. HMBC spectrum of mikanolide

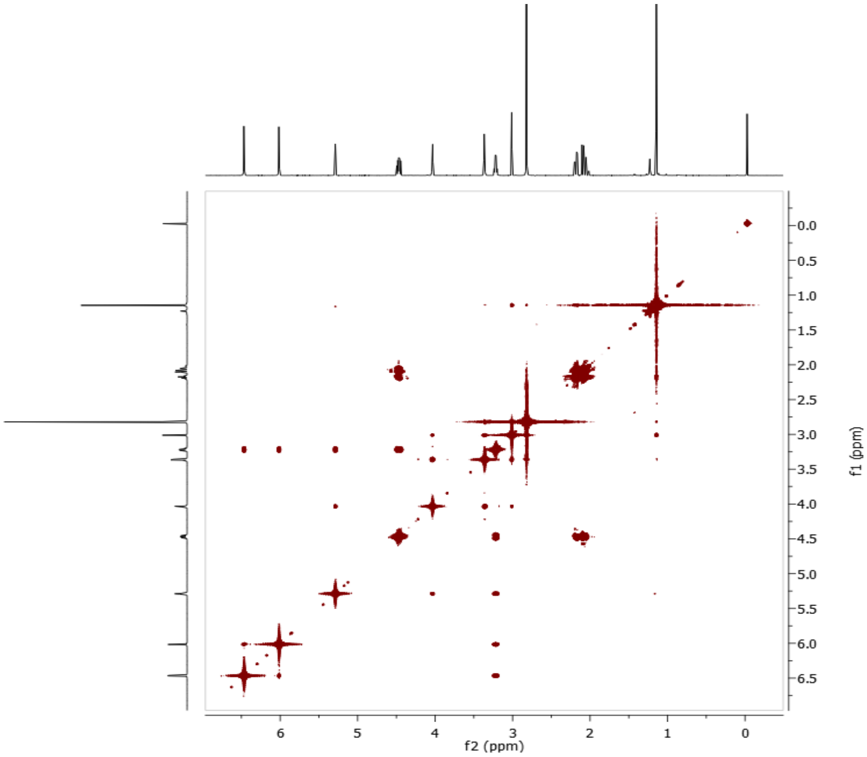

Figure S6. ^{1}H-^{1}H COSY spectrum of mikanolide

2. Chemical structure and spectral data of eupatoriopicrin (compound 2)

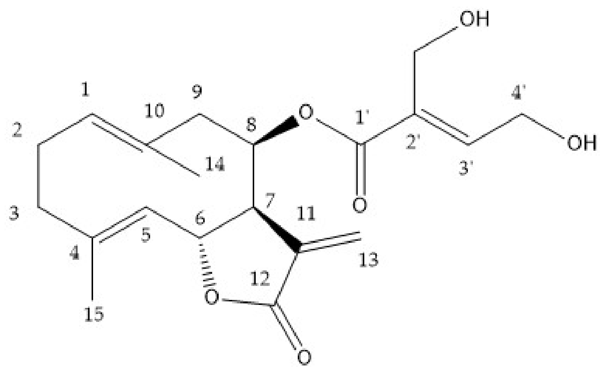

Figure S7. Chemical structure of eupatoriopicrin

IR (ATR), γmáx (cm^{-1}): 1738, 1714 (C=O), 1660 (C=C), 3419 (-OH).

MS (70 eV) m/z (int. rel.): 363 $[M+H^+]$ (0.4%), 362 $[M^+]$, 231 (11.72%), 216 (3.9%), 176 (9.54%), 131 (13.77%), 113 (4.04%), 119 (40.89%), 105 (27.25%), 97 (52.27%) , 95 (100%), 91 (35.3%), 69 (56.88%), 55 (37.74%), 43 (51.56%), 41 (55.06%).

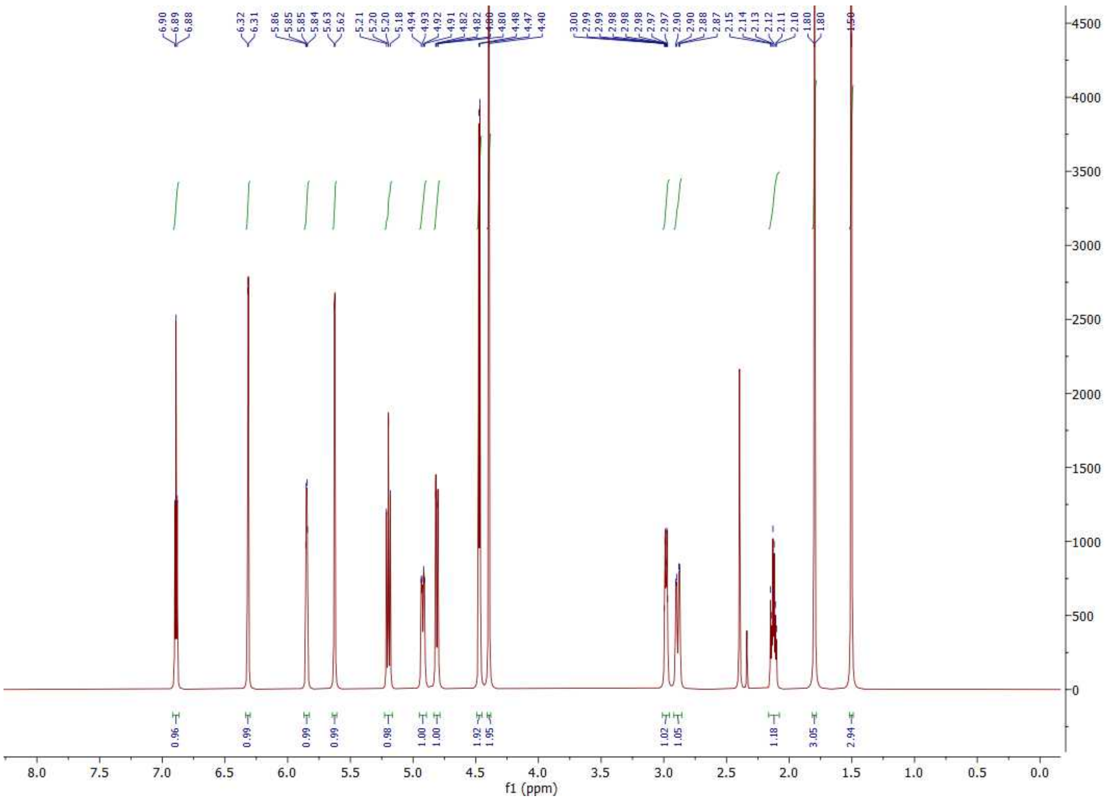

Figure S8. ^{1}H-NMR spectrum of eupatoriopicrin

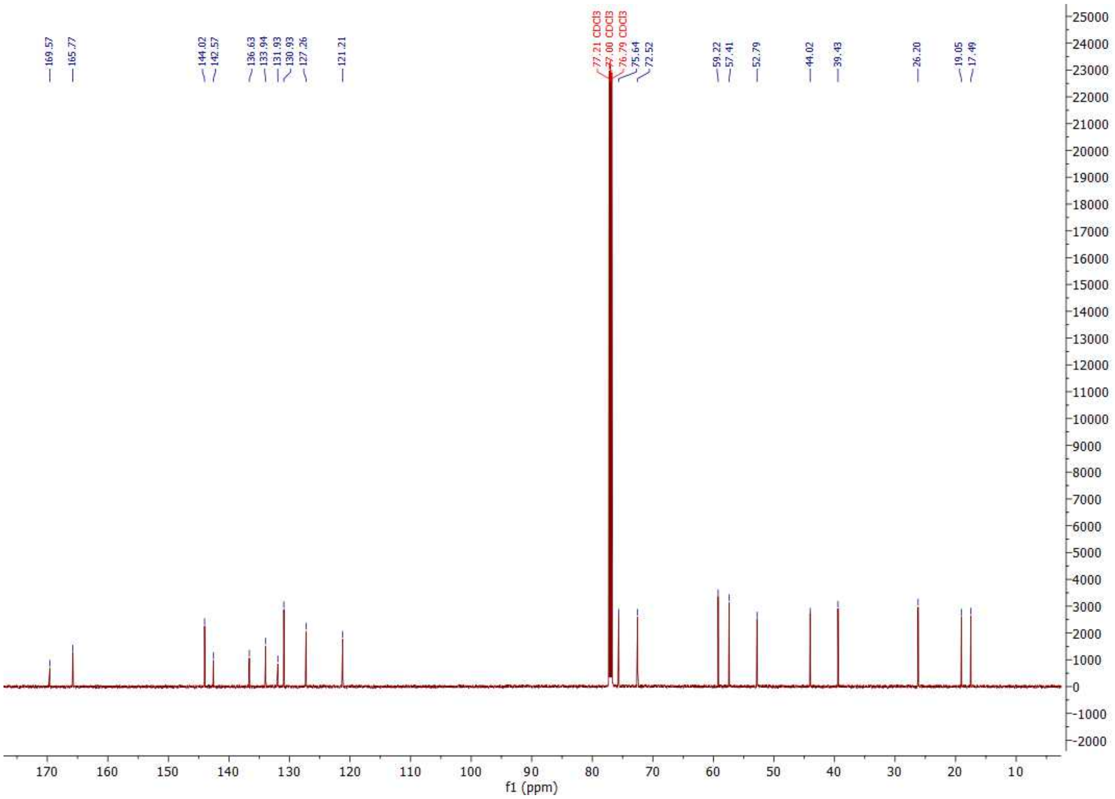

Figure S9. ^{13}C-NMR spectrum of eupatoriopicrin

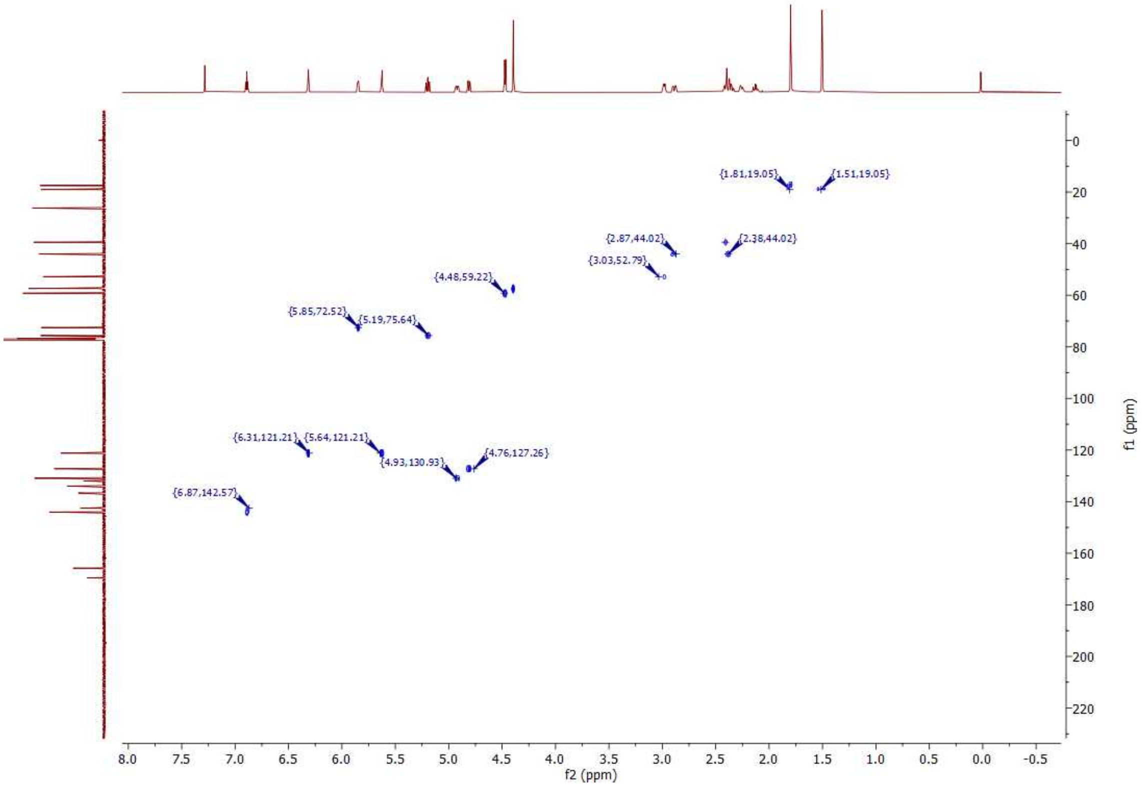

Figure S10. HSQC spectrum of eupatoriopicrin

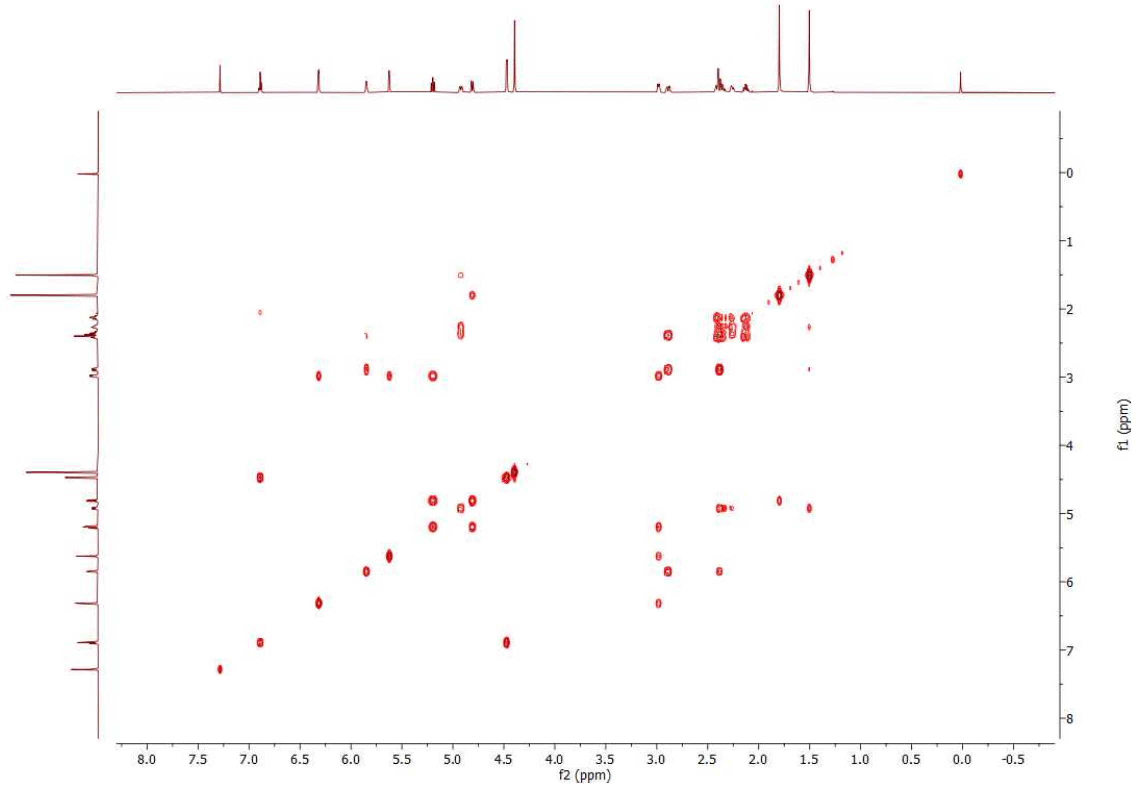

Figure S11. ^1H-^1H COSY spectrum of eupatoriopicrin

3. Chemical structure and spectral data of eupahakonenin B (compound 3)

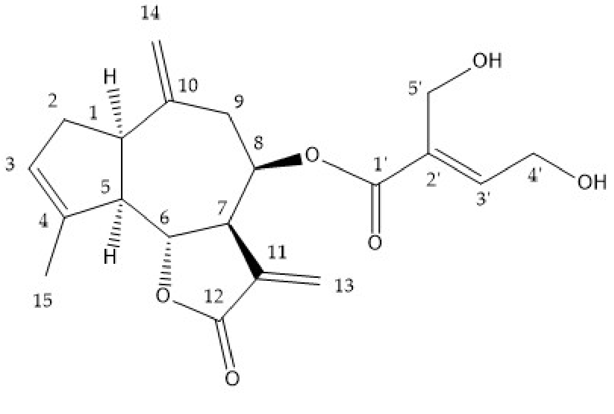

Figure S12. Chemical structure of eupahakonenin B

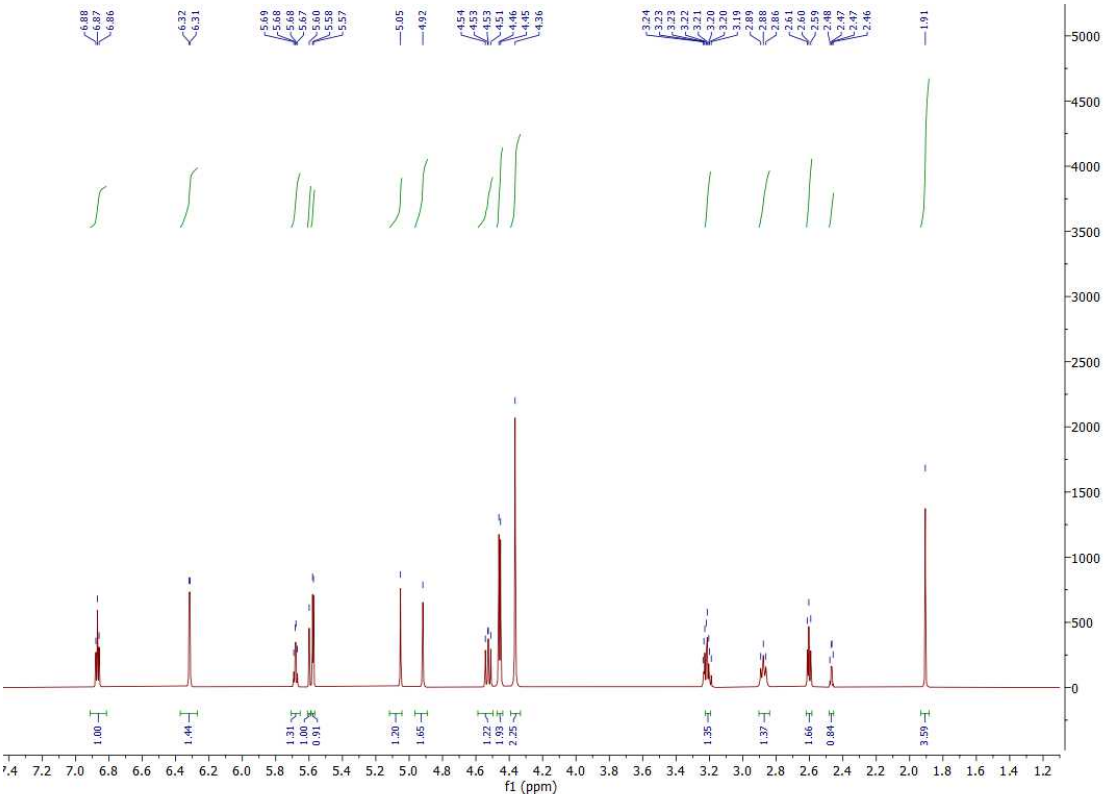

Figure S13. ^{1}H-NMR spectrum of eupahakonenin B

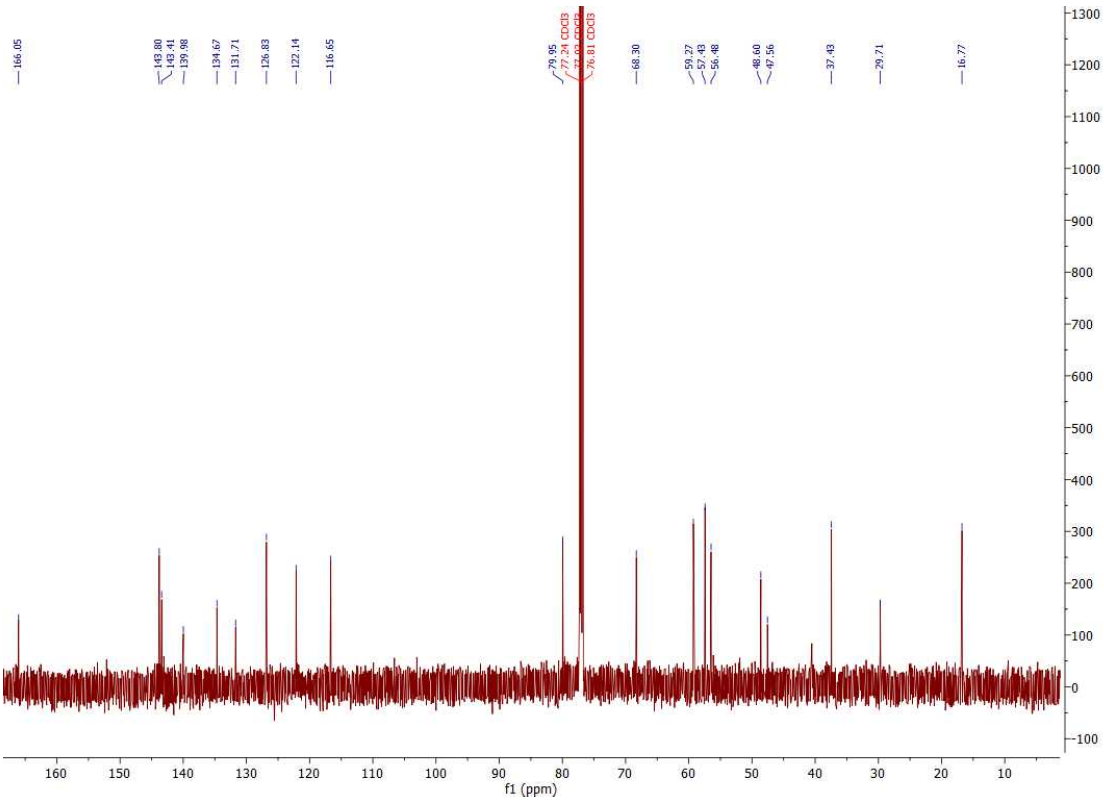

Figure S14. ^{13}C-NMR spectrum of eupahakonenin B

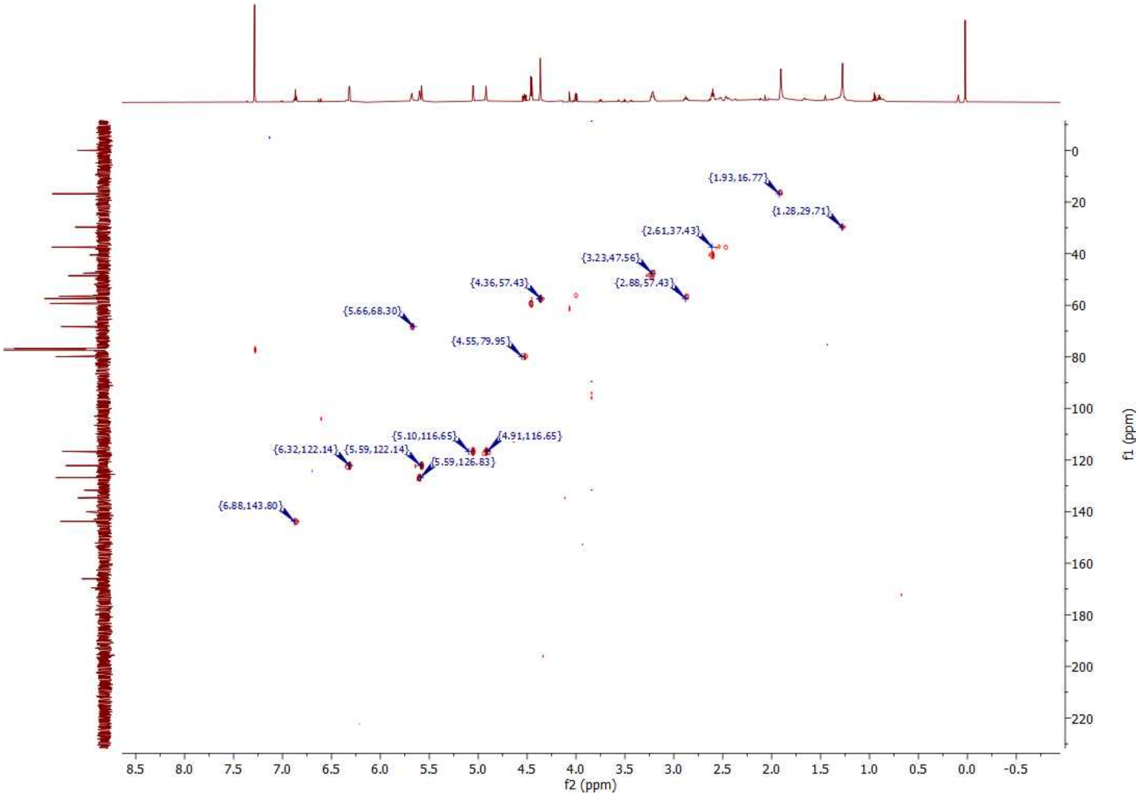

Figure S15. HSQC spectrum of eupahakonenin B

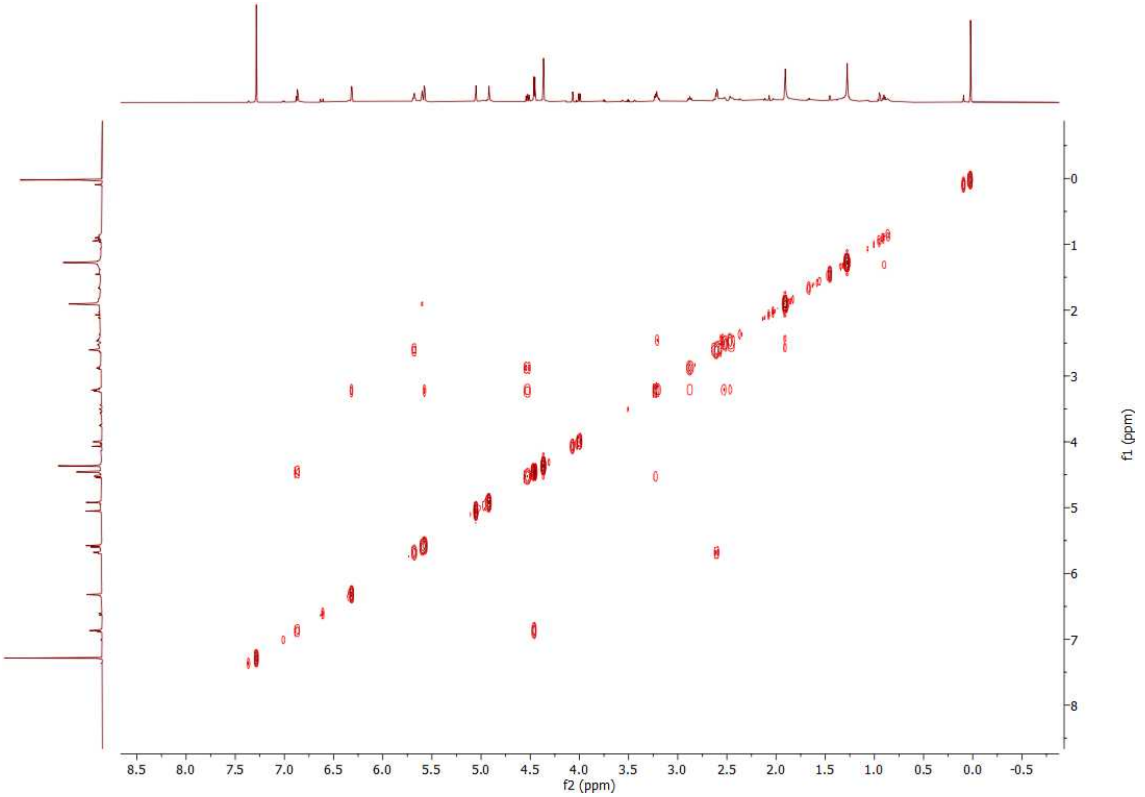

Figure S16. ^{1}H-^{1}H COSY spectrum of eupahakonenin B

4. Chemical structure and spectral data of 2-oxo-8-deoxyligustrin (compound 6)

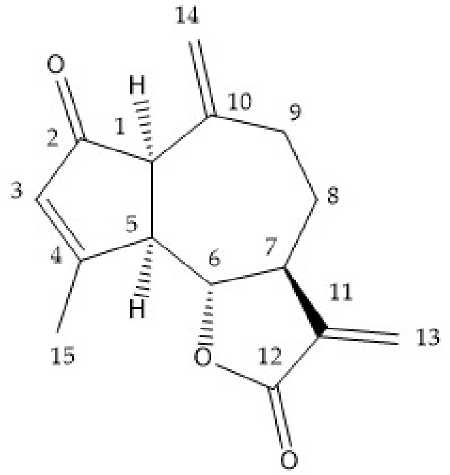

Figure S17. Chemical structure of 2-oxo-8-deoxyligustrin

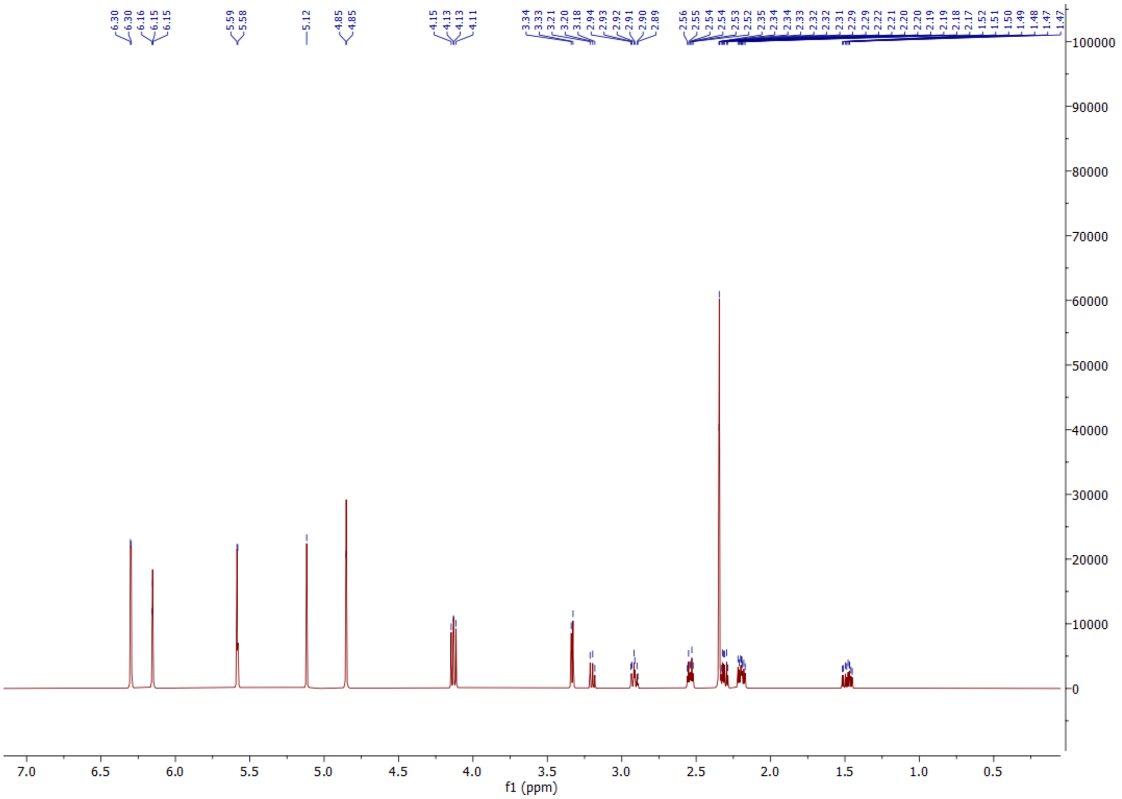

Figure S18. ^{1}H-NMR spectrum of 2-oxo-8-deoxyligustrin

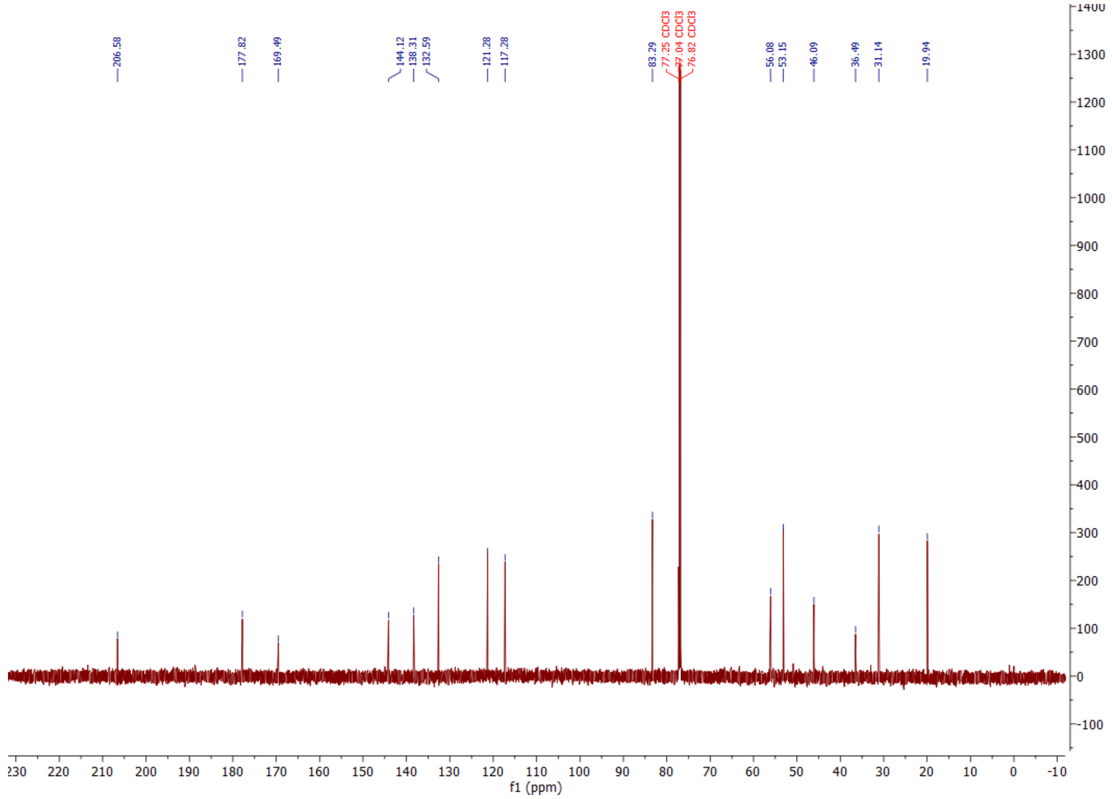

Figure S19. ^{13}C-NMR spectrum of 2-oxo-8-deoxyligustrin

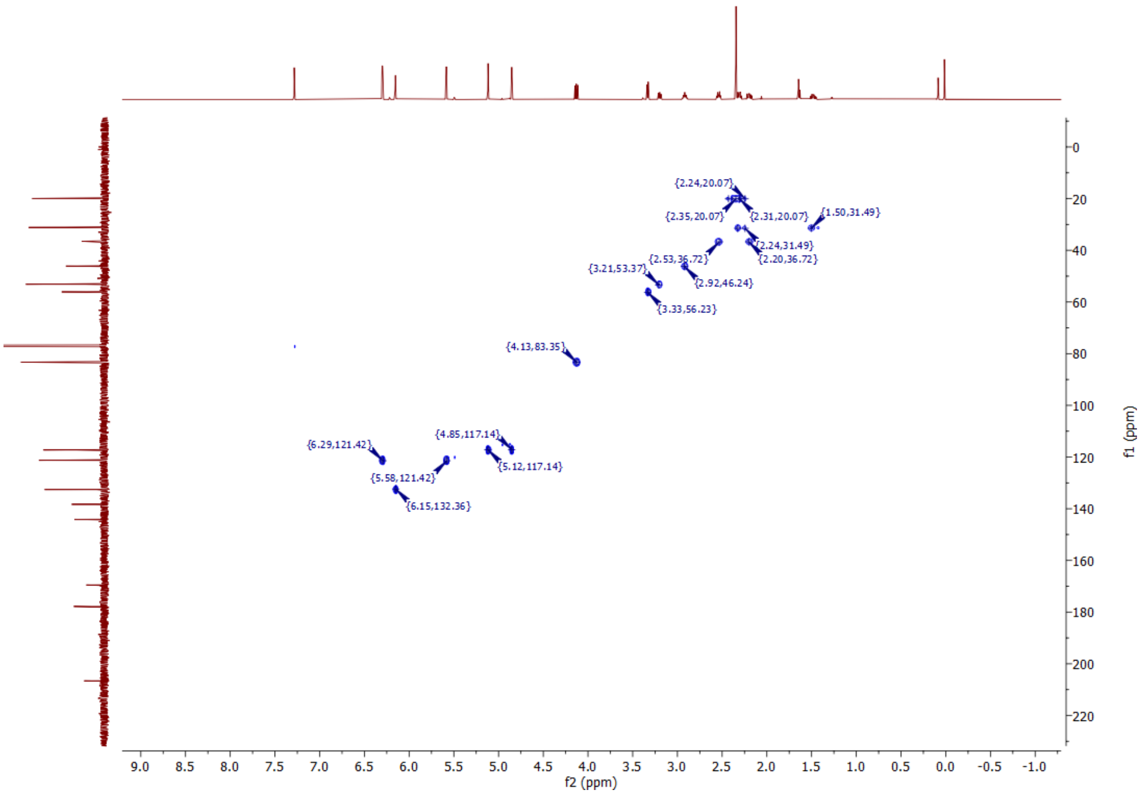

Figure S20. HSQC spectrum of 2-oxo-8-deoxyligustrin

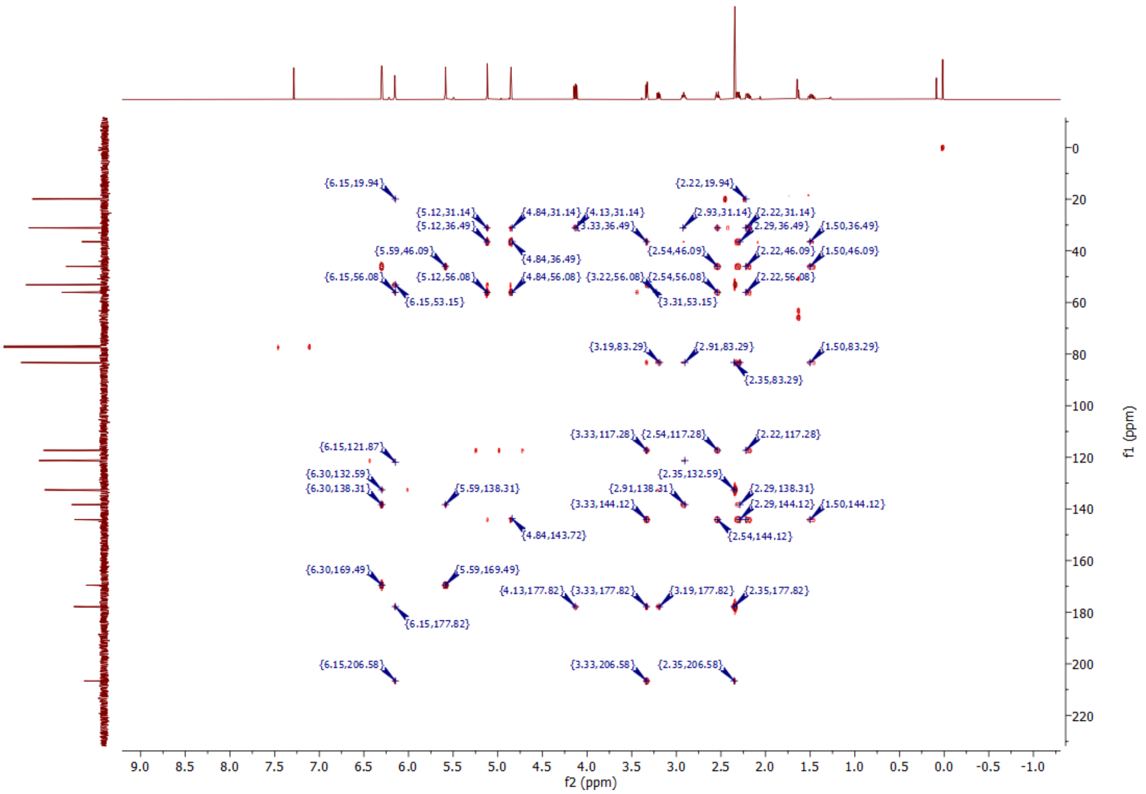

Figure S21. HMBC spectrum of 2-oxo-8-deoxyligustrin

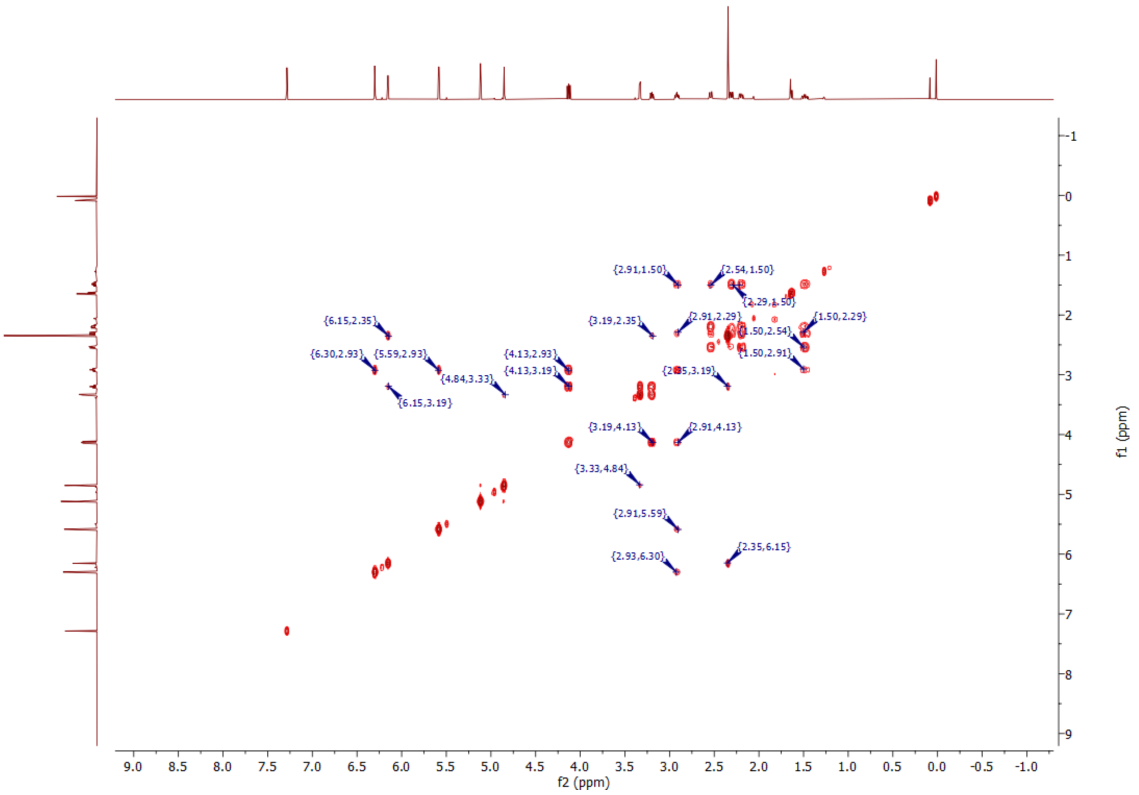

Figure S22. $^{1}H^{1}H$-COSY spectrum of 2-oxo-8-deoxyligustrin

5. Chemical structure and spectral data of eupatorin (compound 13)

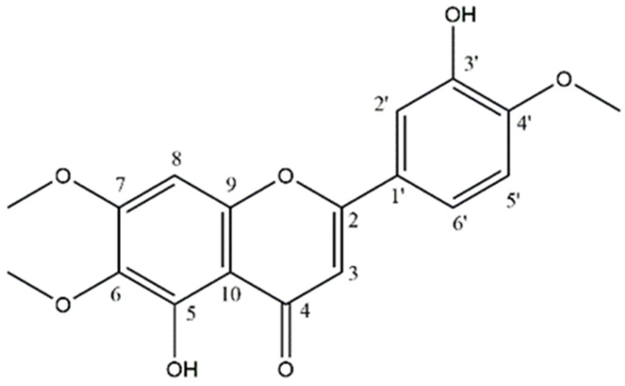

Figure S23. Chemical structure of eupatorin

LC-ESI/MS m/z (rel. int): 345.1096 $[M+H]^+$ (100.0), 367.0923 $[M+Na]^+$ (31.2), 711.1928 $[2M+Na]^+$ (83.2).

^{1}H NMR (600 MHz, $CDCl_3$) δ 12.74 (s, 1H, 5-OH), 7.47 (d, *J* = 2.3 Hz, 1H, H-2′), 7.43 (dd, *J* = 8.5, 2.2 Hz, H, H-6′), 6.95 (d, *J* = 8.5 Hz, 1H, H-5′), 6.57 (s, 1H, H-3), 6.54 (s, 1H, H-8), 5.75 (s, 1H, 3′-OH), 3.98 (s, 3H, 4′-OCH_3), 3.96 (s, 3H, 7-OCH_3), 3.92 (s, 3H, 6-OCH_3).

^{13}C NMR (151 MHz, $CDCl_3$) δ 182.84 (C-4), 163.97 (C-2), 158.92 (C-7), 153.39 (C-5), 153.20 (C-9), 149.73, C-4′), 146.19 (C-3′), 132.79 (C-6), 124.67 (C-1′), 119.27 (C-6′), 112.49 (C-2′), 110.85 (C-5′), 106.34 (C-10), 104.68 (C-3), 90.73 (C-8), 61.02 (6-OCH_3), 56.47 (7-OCH_3), 56.31 (7-OCH_3).

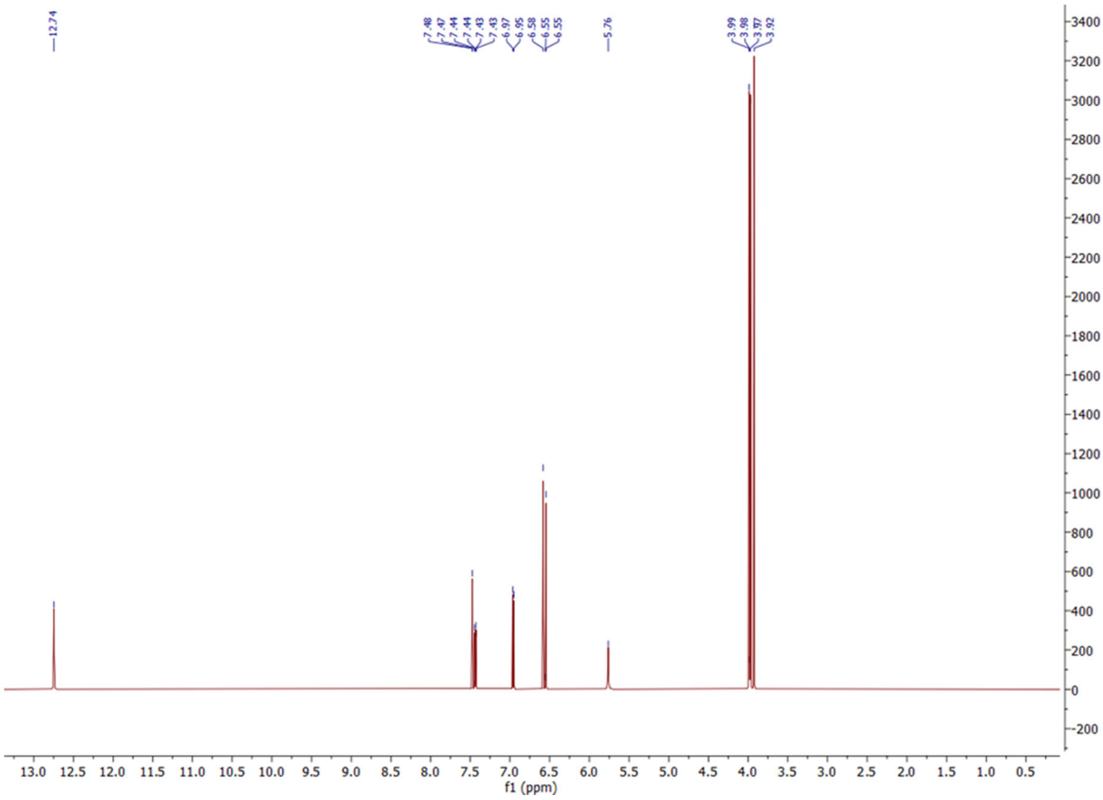

Figure S24. ¹H-NMR spectrum of eupatorin

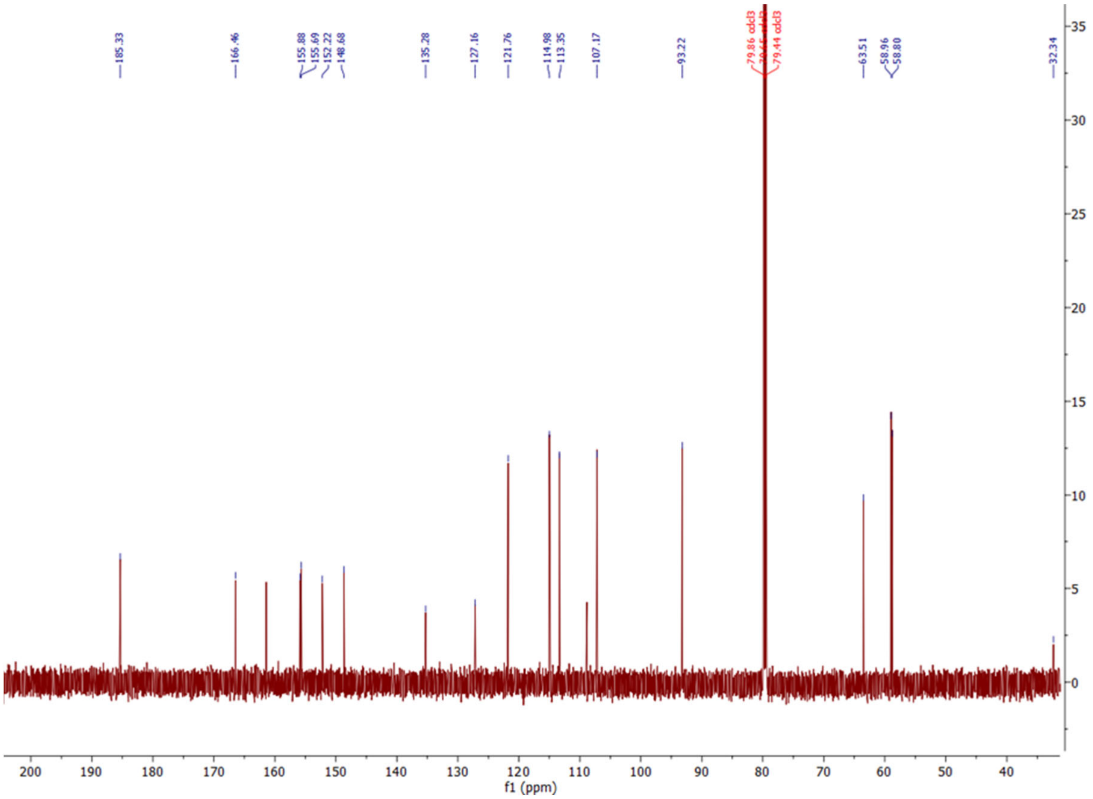

Figure S25. ¹³C-NMR spectrum of eupatorin

6. Chemical structure and spectral data of 5-demethylsinensetin (compound 14)

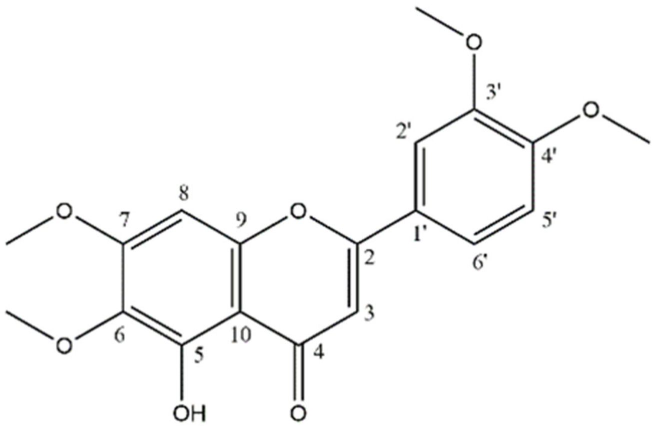

Figure S26. Chemical structure of 5-demethylsinensetin

EI/MS (70 eV) m/z (rel. int): 359.4 [M+H]$^+$ (28.05) 358.4 [M]$^+$ (97.82), 343.2 [M-Me]$^+$ (60.78), 312.4 [M-CO-H_2O]$^+$ (23.89), 83.2 (44.13), 57.2 (61.40), 55.2 (72.06), 43.2 (100.00), 41.2 (50.14).

^{1}H NMR (600 MHz, $CDCl_3$) δ 12.78 (s, 1H, 5-OH), 7.56 (dd, *J* = 8.5, 2.1 Hz, 1H, H-6′), 7.37 (d, *J* = 2.1 Hz, 1H, H-2′), 7.01 (d, *J* = 8.5 Hz, 1H, H-5′), 6.63 (s, 1H, H-3), 6.58 (s, 1H, H-8), 4.02 (s, 3H, OCH_3), 4.01 (s, 3H, OCH_3), 4.00 (s, 3H, OCH_3), 3.96 (s, 3H, OCH_3).

^{13}C NMR (125 MHz, $CDCl_3$) δ 182.63 (C-4), 163.99 (C-2), 158.75 (C-7), 153.24 (C-9), 153.08 (C-5), 152.30 (C-3'), 149.34 (C-4'), 132.67 (C-6), 123.81 (C-1'), 120.09 (C-6'), 111.18 (C-5'), 108.80 (C-2'), 106.17 (C-10), 104.51 (C-3), 90.60 (C-8), 60.89 (6-OMe), 56.36 (7-OMe), 56.16 (3'-OMe), 56.13 (4'-OMe).

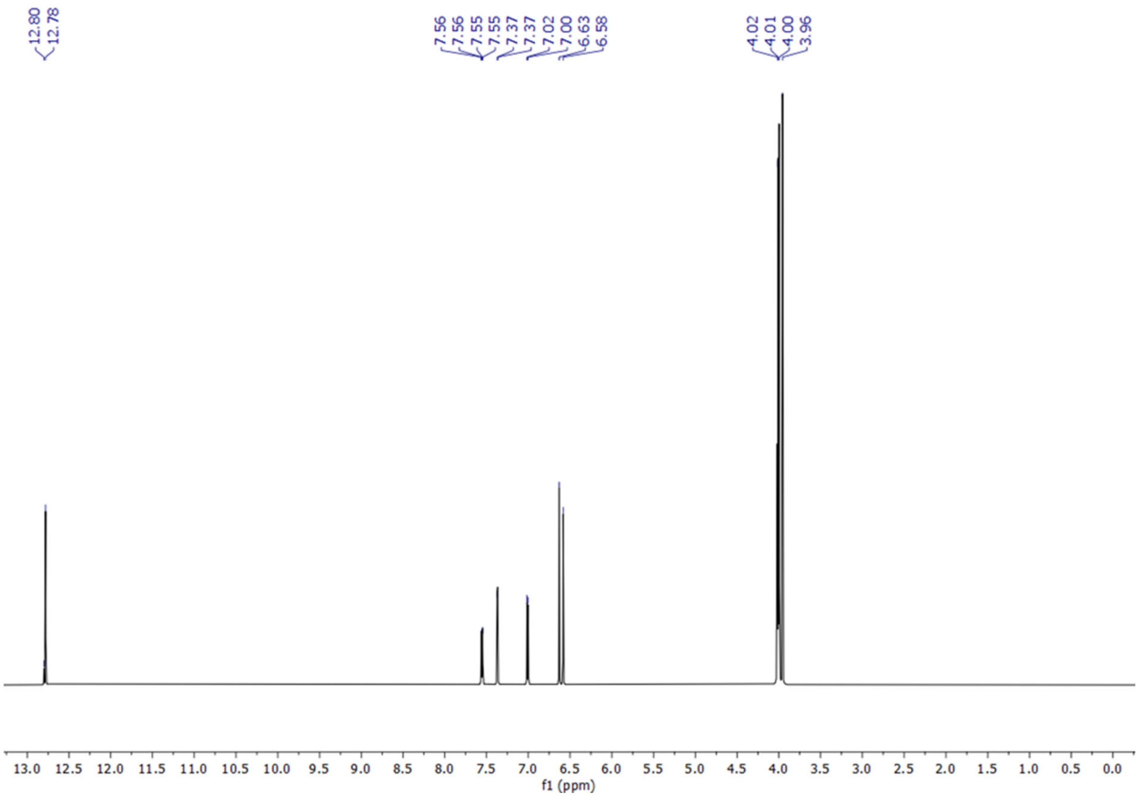

Figure S27. ^{1}H-NMR spectrum of 5-demethylsinensetin

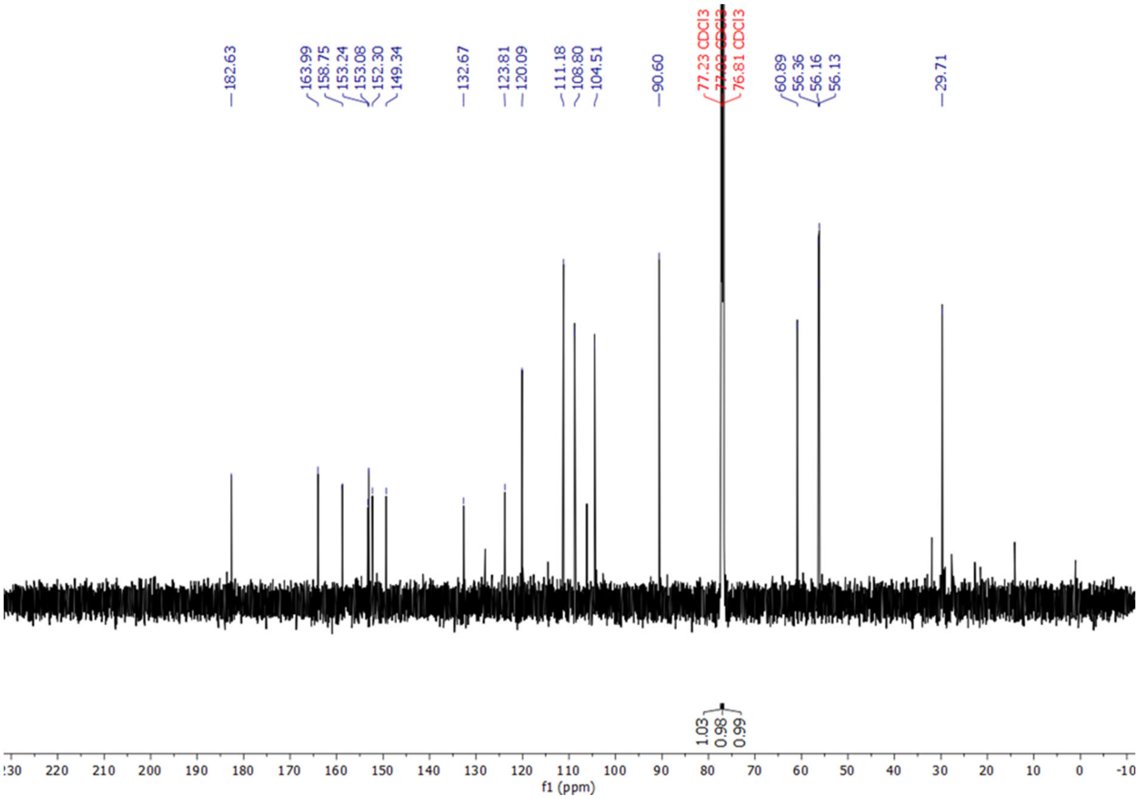

Figure S28. ^{13}C-NMR spectrum of 5-demethylsinensetin

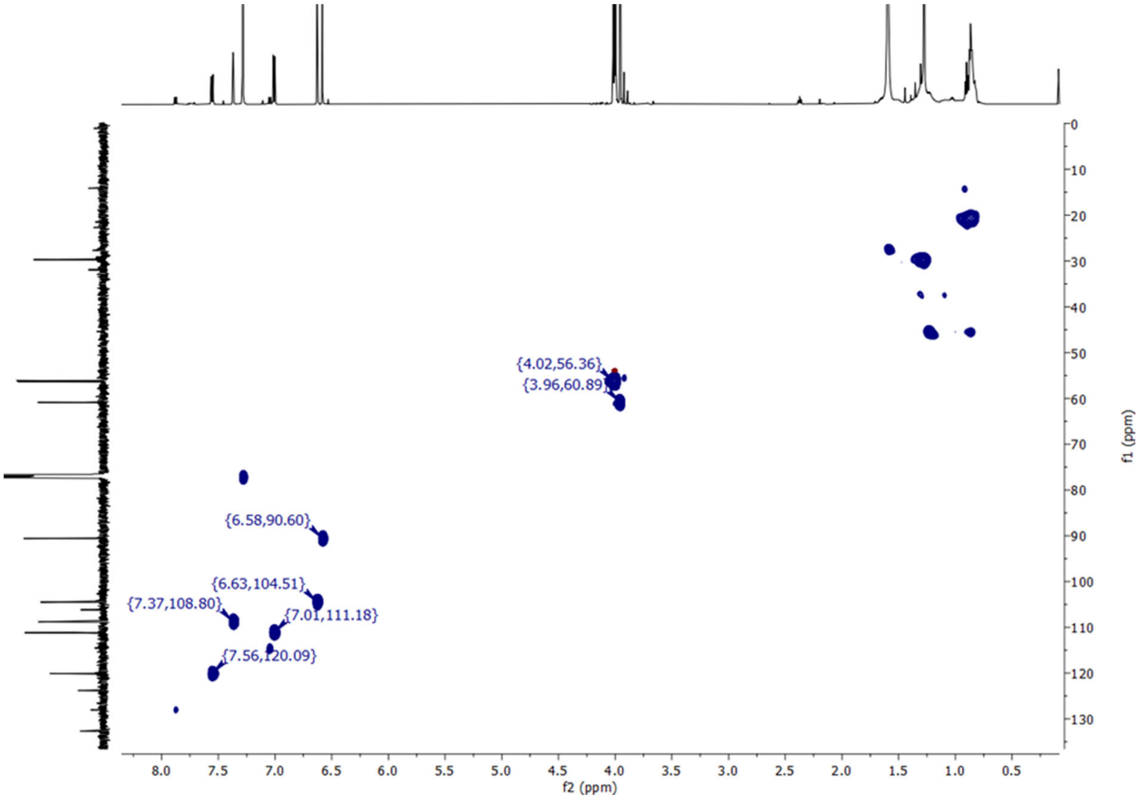

Figure S29. HSQC spectrum of 5-demethylsinensetin

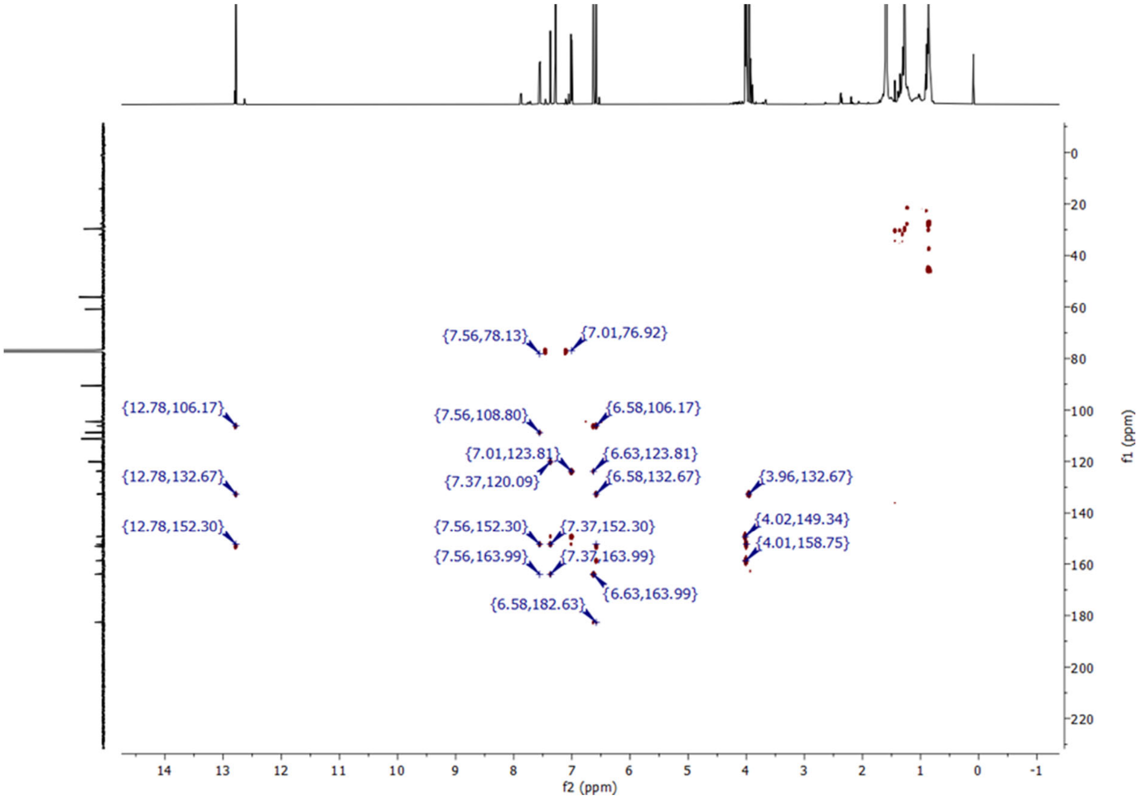

Figure S30. HMBC spectrum of 5-demethylsinensetin

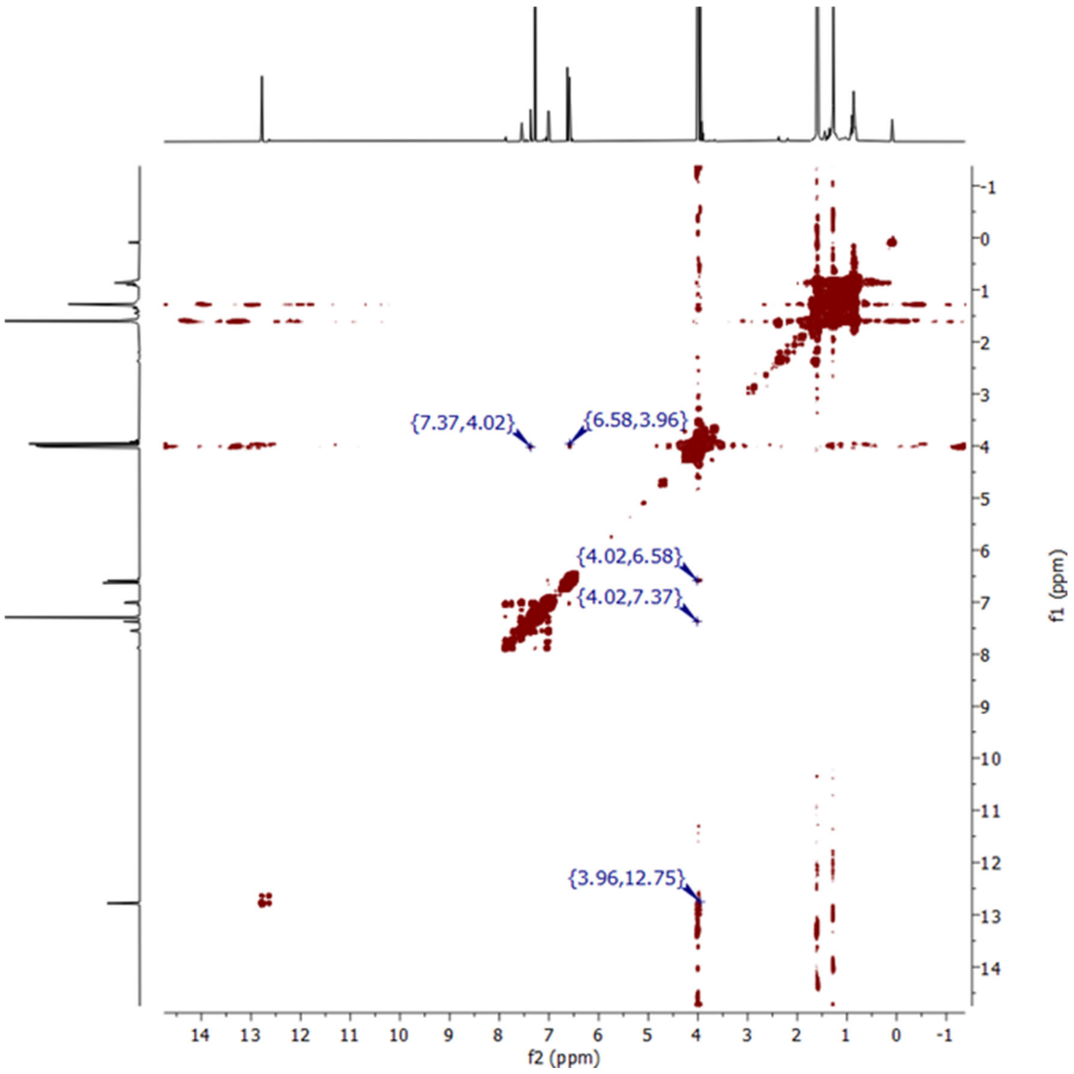

Figure S31. ^{1}H-^{1}H COSY spectrum of 5-demethylsinensetin

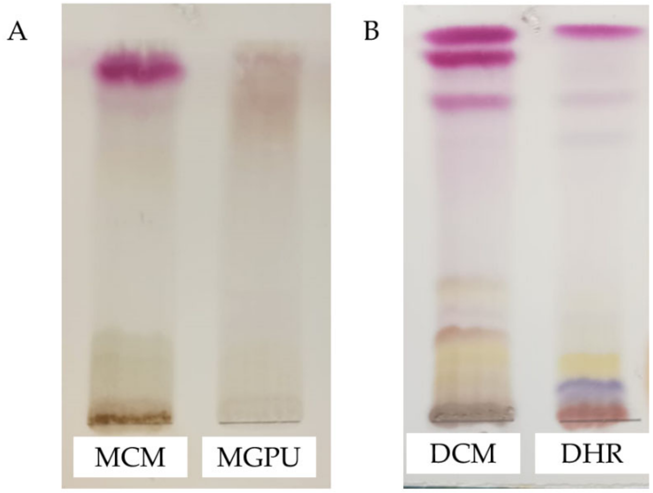

Figure S32. TLC analysis of methanolic and dichloromethane extracts MGPU, MCM, DHR and DCM. (A) MCM and MGPU (Chromatographic system: Stationary phase: Silicagel F_{254}, Mobile phase: ETAC:MEOH (10:5), spray reagent: Anisaldehyde-sulphuric acid; (B) DCM and DHR (Chromatographic system: Stationary phase: Silicagel F_{254}, Mobile phase: HX:ETAC (1:1), spray reagent: Anisaldehyde-sulphuric acid.
